# Network-Based Identification of Altered Stem Cell Pluripotency and Calcium Signaling Pathways in Metastatic Melanoma

**DOI:** 10.3390/medsci6010023

**Published:** 2018-03-08

**Authors:** Ben-Hur Neves de Oliveira, Carla Dalmaz, Fares Zeidán-Chuliá

**Affiliations:** 1 Departamento de Bioquímica, Instituto de Ciências Básicas da Saúde, Universidade Federal do Rio Grande do Sul (UFRGS), Porto Alegre, RS 90035-003, Brazil; ben.neves@ufrgs.br (B.-H.N.d.O.); cdalmaz@ufrgs.br (C.D.); 2 Departamento de Ciencias Biomédicas Básicas, Facultad de Ciencias Biomédicas y de la Salud, Universidad Europea de Madrid, 28670 Villaviciosa de Odón, Spain; 3 Faculty of Medicine, University of Turku, FI-20520 Turku, Finland

**Keywords:** pluripotency, stemness, calcium, malignancy, biomarker, systems biology

## Abstract

Malignancy of cancer has been linked to distinct subsets of stem-like cells, the so-called cancer stem cells (CSCs), which persist during treatment and seem to lead to drug-resistant recurrence. Metastatic spread of cancer cells is one of the hallmarks of malignancy and contributes to most human melanoma-related deaths. Recently, overlapping groups of proteins and pathways were shown to regulate stem cell migration and cancer metastasis, raising the question of whether genes/proteins involved in stem cell pluripotency may have important implications when applied to the biology of cancer metastasis. Furthermore, it is well known that ion channels and receptors, particularly those responsible for calcium (Ca^2+^) signal generation, are critical in determining the cellular fate of stem cells (SCs). In the present study, we searched for evidence of altered stem cell pluripotency and Ca^2+^ signaling-related genes in the context of melanoma metastasis. We did this by using network analysis of gene expression in tissue biopsies from three different independent datasets of patients. First, we created an in silico network model (“STEMCa” interactome) showing the landscape of interactions between stem cell pluripotency and Ca^2+^ signaling-related genes/proteins, and demonstrated that around 51% (151 out of 294) of the genes within this model displayed significant changes of expression (False Discovery Rate (FDR), corrected *p-*value < 0.05) in at least one of the datasets of melanoma metastasis when compared with primary tumor biopsies (controls). Analysis of the properties (*degree* and *betweenness*) of the topological network revealed 27 members as the most central hub (HB) and nonhub-bottlenecks (NH-B) among the 294 genes/proteins of the whole interactome. From those representative genes, *CTNNB1*, *GNAQ*, *GSK3B*, *GSTP1*, *MAPK3*, *PPP1CC*, *PRKACA*, and *SMAD4* showed equal up- or downregulation (corrected *p-*value < 0.05) in at least 2 independent datasets of melanoma metastases samples and *PTPN11* showed upregulation (corrected *p-*value < 0.05) in three of them when compared with control samples. We postulate that altered expression of stem cell pluripotency and Ca^2+^ signaling pathway-related genes may contribute to the metastatic transformation, with these central members being an optimal candidate group of biomarkers and in silico therapeutic targets for melanoma metastasis, which deserve further investigation.

## 1. Introduction

During the last 20 years of cancer research, the so-called cancer stem cell (CSC) theory has generated and still generates debates among the scientific community as to the critical participation of these cells in tumorigenesis, regarding their main characteristics to (i) initiate, (ii) maintain, (iii) re-grow diverse tumors, and (iv) give rise to drug-resistant recurrence [1,2]. The molecular mechanisms of cancer metastasis are still under study, but metastasis is known to develop from a small population of cancer cells within the primary tumor that is capable of entering and surviving in the circulation and then exiting to re-grow in a distant tissue [3]. The way in which the CSC theory may fit with the general scheme of metastatic potential is not well defined to date.

Melanoma is a neoplasm stemming from melanocytes or the cells that develop from melanocytes. Since the increase in popularity of the CSC theory, single stem cell markers, including ABCB5, CD20, CD133, CD271, and ALDH1A, have been reported in the literature to correlate with melanoma malignancy [4]. These studies invite further investigation into the potential role of stem cell pluripotency-related genes/proteins in tumor progression and, potentially, metastatic transformation. Considering that stem cells (SCs) have the capability to self-renew and to differentiate into more specialized cells [5], the signaling pathways governing their properties are also the subject of intense research. For instance, ion channels and its receptors, particularly those responsible for calcium (Ca^2+^) signal generation, are critical for determining the cellular fate and differentiation of SCs. It is, therefore, plausible that transcriptional changes in members of the Ca^2+^ signaling pathway may trigger pathological events such as tumorigenesis [6,7]. However, only a limited number of studies have explored the putative link between intracellular Ca^2+^ and the metastasis of human cancer cells. For example, a recent study (2016) by Rizaner and colleagues observed spontaneous Ca^2+^ oscillations in a proportion of strongly metastatic human prostate and breast cancer cells [8].

The biggest challenge for cancer researchers probably resides in the discovery of robust markers of survival and metastatic potential, and to provide early diagnosis and treatment for patients. The detection and follow-up of disease markers are nowadays based on solitary proteins and most of the time this approach is unreliable. Wider and more powerful systems than conventional pathology-based data (i.e., immunohistochemical)—capable of dealing with and analyzing increasing amounts of information—are needed, such as bioinformatics and systems biology tools [9]. For example, by integrating additional information into biological pathways or into protein–protein interaction (PPI) network models, we could find modular biomarkers that integrate multiple genes with potential interactions, which could lead to more accurate and reproducible prognostic predictions and better comprehension of disease pathogenesis [10]. In fact, our group has successfully applied network-based approaches and systems biology analyses in the context of diverse pathologies such as autism, Alzheimer’s disease, and periodontal inflammation [11,12,13,14,15,16,17].

**Figure 1:**
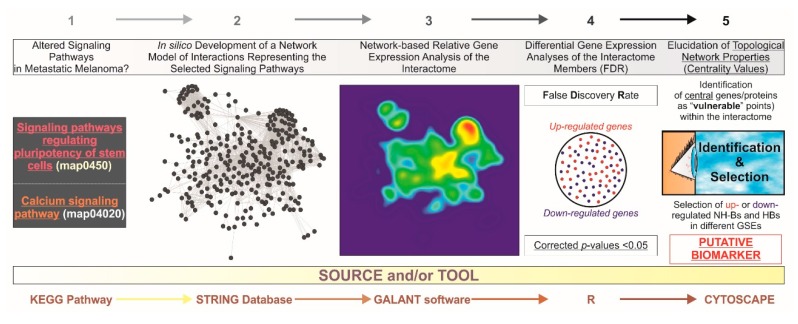
General abstract workflow summarizing the criteria and tools utilized for the present study. Two maps from the KEGG PATHWAY Database corresponding to “Signaling pathways regulating pluripotency of stem cells” (map04550) and “Ca^2+^ signaling pathway” (map04020) were selected. Thereafter, an interactome was developed for integrating these maps into a single gene/protein interaction network model by the STRING V10.0 database (http://string-db.org/). With GALANT (GrAph LANdscape VisualizaTion), a Cytoscape (http://cytoscape.org/) plugin for visualizing data as functional landscapes projected onto biological networks, we projected numerical data from three different datasets extracted from the GEO database (http://www.ncbi.nlm.nih.gov/geo/). The 2D views correspond to the relative gene expression on the newly-developed interactome. This tool creates three smoothed data plots that resemble the network model layout. Differential gene expression analysis was performed by using the R environment, *limma* package (http://www.R-project.org/). The *p*-values were adjusted for multiple comparisons by calculating false discovery rates (FDR) to avoid potential false positives. By using Cytoscape, further identification of “vulnerable” points within the in silico model upon the elucidation of centrality values and the selection of those central (hub (HB) and nonhub-bottlenecks (NH-B) up or downregulated genes suggest candidate diagnostic biomarkers and also potential molecular targets for therapeutics. GSEs: unique and stable GEO accession number assigned for each series record or group of related samples.

In this study, we created an integrated in silico network model of interactions to seek evidence of altered stem cell pluripotency and Ca^2+^ signaling pathways in microarray samples from three independent datasets of patients (Figure 1). By using a system biology-based approach, we searched for de-regulated central genes, at the transcriptional level, which could represent putative biomarkers for melanoma metastasis. Based on our findings, we hypothesize that aberrant signaling of stem cell pluripotency and Ca^2+^ pathways may account for metastatic transformation, and specifically propose nine representative central genes from our whole network model as an optimal candidate group of biomarkers for human metastatic melanoma.

## 2. Material and Methods

### 2.1. Development of the Gene/Protein Interaction Network Model, Data Acquisition, and Processing

For developing an integrated in silico network model (“STEMCa”), we first retrieved all the genes annotated as members of the KEGG PATHWAY Database entries x and y, which were, respectively, “Signaling pathways regulating pluripotency of stem cells” (map04550) and “Ca^2+^ signaling pathway” (map04020) (Supplementary Table S1). The human interactome (i.e., annotated interactions between human proteins) was downloaded from the STRING V10.0 database (http://string-db.org/) [18] in a text file and read into the R programming environment where scripts were written to generate our PPI network. We used only interactions reported by STRING as being from either “database” or “experimental evidence” and also with a minimum score of 0.400 (medium confidence). The basic steps for network generation were as follows: (i) proteins encoded by the KEGG gene list were used as input to build the PPI network with the set parameters; (ii) the algorithm searched for disconnected nodes on the network (i.e., proteins with no reported interaction with the main network); (iii) if there were disconnected nodes, the algorithm would hunt for neighboring proteins or connectors (CONs) that could be included in the network with the purpose of joining disconnected nodes to the main network; (iv) if two or more nodes were able to join a set of disconnected nodes to the main network, then the one with the highest degree was selected; and finally, (v) the algorithm stopped only when there were no more nodes that could be added. The rationale behind this approach was to build a fully connected PPI network model from a list of proteins with the least possible interference from outsiders (proteins that were not originally inputs) and without losing network density connection.

### 2.2. Relative Gene Expression Network Visualization and Microarray Analysis

For our expression meta-analysis, we searched for cancer microarray raw data on the GEO database (http://www.ncbi.nlm.nih.gov/geo/) using the following as keywords: “melanoma” and “metastasis” or “metastatic melanoma” in *Homo sapiens*. Selected GSEs (unique and stable GEO accession number assigned for each series record or group of related samples) were GSE8401 (*N* = 83), GSE46517 (*N* = 104), and GSE15605 (*N* = 58). The database is publicly available, and data were originally contributed by Xu et al. [19], Kabbarah et al. [20], and Raskin et al. [21], respectively. Experimental assays are explained in detail in these original publications, including the selection criteria of the patients. Raw data normalization technique was chosen based on each dataset platform. Differential gene expression analysis was performed through the implemented functions for R in the *limma* package, which uses linear models to calculate moderated t-statistics for microarray data. The *p*-values were adjusted for multiple comparisons by calculating false discovery rates (FDR) [22]. Only genes with corrected *p*-values < 0.05 (FDR) were considered as differentially expressed (Supplementary Table S2). Log-fold changes for each of the encoding genes of the proteins on the in silico model were exported from R into text files and plotted over the PPI network (metastatic vs. primary melanoma samples) by using the plugin GALANT for Cytoscape (Gaussian function sigma set to 0.04), which builds functional landscapes onto biological networks [23].

### 2.3. Elucidation of the Topological Network Properties (Degree or Connectivity and Betweenness)

Elucidation of the topological network properties [24] such as *degree* (also known as *connectivity*) and *betweenness* centralities (Supplementary Table S2) was performed by using the Network Analyzer plugin from the Cytoscape software. Values of centralities above one standard deviation (+1 SD) of the mean were selected to identify central nodes within the interactome. *Degree* gives information about the local topology of each node by summing the number of its adjacent nodes. Nodes with high values of *degree* over the threshold values are known as hubs. *Betweenness* provides values about how frequently the shortest path connects each pair of nodes through a third given node. Therefore, *betweenness* provides information about the influence of a node over the spread of information throughout the interactome. Nodes with values of *betweenness* over the thresholds are named as “bottlenecks” (nonhub- and hub-bottlenecks are represented by NH-Bs and HBs, respectively).

## 3. Results

### 3.1. “STEMCa” Is an Integrative Network Model of Interactions for Stem Cell Pluripotency and Ca^2+^ Signaling Pathways

**Figure 2:**
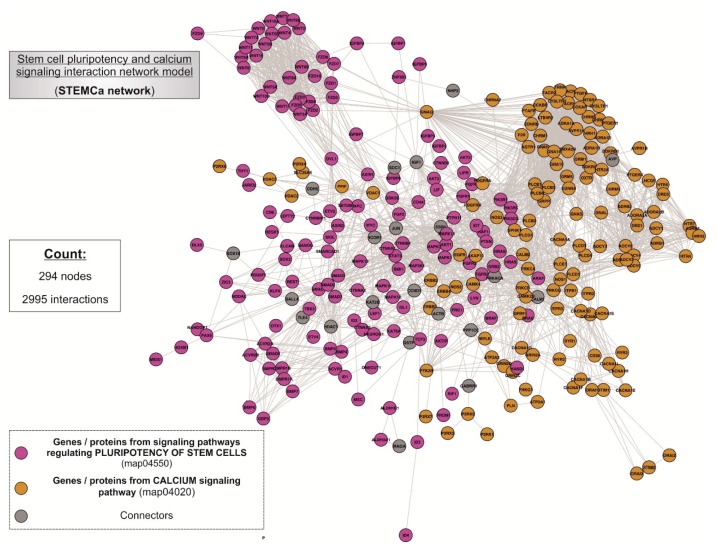
“STEMCa” interactome. General landscape of interactions between the genes/proteins of stem cell pluripotency (map04550) and calcium signaling (map04020) interaction network model. The present in silico network model was developed by using the STRING V10.0 database resource search tool, exclusively using “experiments” and “databases” as input options for active prediction methods and a minimum confidence score of 0.400 (medium confidence).

Two pathways, the stem cell pluripotency (map04550) and Ca^2+^ signaling (map04020) (Supplementary Table S1), were integrated within the interactome (Figure 2), being composed of 294 nodes interconnecting through 2995 interactions. In the case of non-existing direct crosstalk through common members from both signaling pathways, it was possible to search for common neighboring nodes corresponding to additional genes/proteins or CONs to members of both pathways by using mathematical algorithms in the R environment (see Section 2). Our model included 21 CONs within the “STEMCa” interactome.

### 3.2. Network-Based Analyses of Stem Cell Pluripotency and Ca^2+^ Signaling Pathways Reveal Transcriptional Changes in Independent Datasets of Human Metastatic Melanoma

**Figure 3:**
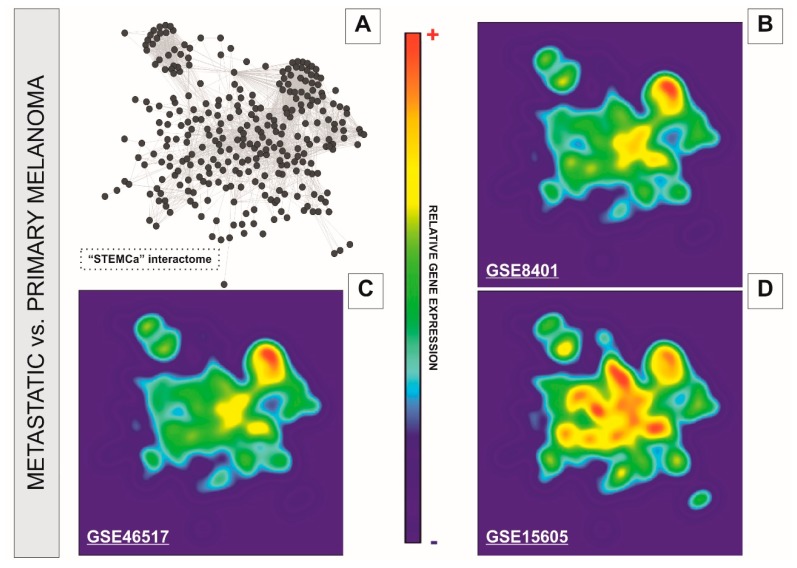
GALANT plots over the in silico model “STEMCa” in melanoma metastasis from three independent sample sources from GEO. The figure represents the relative gene expression over the “STEMCa” interactome (**A**) in melanoma metastasis vs. primary melanoma analyzed in (**B**) GSE8401, (**C**) GSE46517, and (**D**) GSE15605 independent microarray samples, by using the GALANT software, which is an open-source application able to build landscape maps of gene expression networks.

We performed a systems level analysis of potential transcriptional changes in genes belonging to SC pluripotency and Ca^2+^ signaling pathways in metastatic melanomas. The “STEMCa” interactome (Figure 3A) was subjected to a relative gene expression analysis profile by using the GALANT software, which plotted the expression mean values derived from metastatic melanoma over the expression of primary tumor samples in the network model, presenting it as two-dimensional GALANT-generated landscapes graded by color (Figure 3B–D). The relative gene expression levels were color-coded with warm colors (yellow-red) being representative of higher expression whereas cold (green-blue) colors represent lower gene expression when compared to control samples. The 2D GALANT plots revealed equal landscape patterns of de-regulated relative gene expression in SC pluripotency and Ca^2+^ signaling pathways when metastatic melanomas were compared to primary tumors in three independent datasets (GSE8401, GSE46517, and GSE15605) of microarray samples (Figure 3B–D). Differential expression analysis of the “STEMCa” interactome revealed significant transcriptional changes in metastatic melanoma. In particular, 151 (~51%), 63 (~22%), and 14 (~5%) out of 294 genes from “STEMCa” interactome were differentially expressed (corrected *p*-values < 0.05; FDR) in the tissue samples from one, two, or three independent microarray datasets, respectively (Supplementary Table S2). At this stringent FDR, none of these changes were expected to be a false positive. Of note, in all cases, these genes followed the same pattern of de-regulation (up or downregulation) when the datasets were compared (Supplementary Table S2 and Figure 2). The observed changes may account for the metastatic transformation of human melanoma with consequent alterations in patient survival.

### 3.3. Elucidation of Key Hub Genes/Proteins within the Integrative “STEMCa” Interactome Suggests a Group of Candidate Biomarkers for Metastatic Melanoma

**Figure 4:**
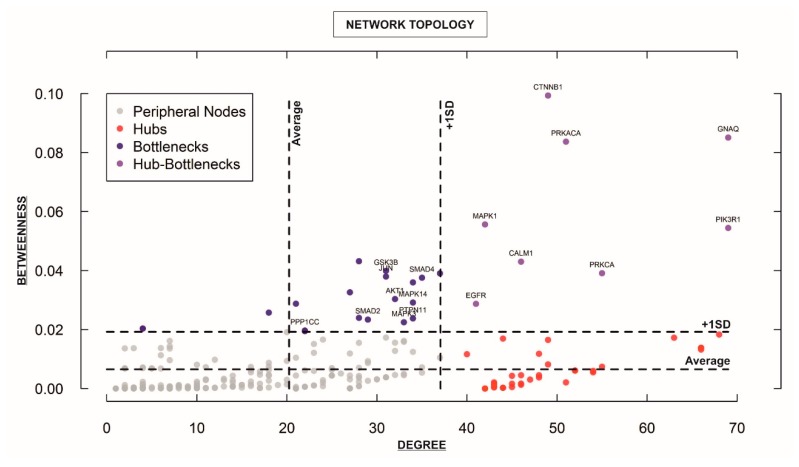
Analysis of the topological properties (*betweenness* vs. *degree*) of genes/proteins belonging to the “STEMCa” interactome. The figure identifies only central nodes, hub-bottlenecks and nonhub-bottlenecks with values above +1 SD of the mean, which are differentially expressed (corrected *p*-values < 0.05) in at least one GSE, which is the unique and stable GEO accession number assigned for each series record or group of related samples, it is the ID from GEO for the analyzed datasets from the re-analyzed cohorts (GSE8401, GSE46517, and GSE15605).

For characterizing the newly-developed interactome, we calculated the topological network properties or centrality values (Supplementary Table S2), known as *degree* or *connectivity* and *betweenness*. These values reveal the most central nodes (genes/proteins) in a network [14,15,16]. Central nodes (NH-B and HB) mark the points of vulnerability within the network of interactions and any variations in these members (e.g., up or downregulation of a given gene) could be considered to trigger more intense changes in the rest of the interactome (e.g., “STEMCa”) than those of non-central or less central nodes. Therefore, one could consider that any gene/protein identified as NH-B or HB may represent one putative biomarker [16], especially if those showed the same qualitative up or downregulation in gene expression (corrected *p*-values < 0.05; FDR) in at least two independent datasets of microarray samples. After calculating centrality values for the 294 “STEMCa” gene/protein members (Supplementary Table S2), we identified 27 central nodes (Figure 4), 19 NH-Bs (values of *betweenness* above one standard deviation of the mean), and 8 HBs (values of *betweenness* and *degree* above one standard deviation of the mean). Respectively, 11, 5, and 1 gene(s) out of 19 NH-Bs showed the same qualitative (up or downregulation) differential gene expression (corrected *p*-values < 0.05; FDR) in these tissue samples from one, two, or even three independent microarray datasets. Eight and 3 out of 8 HBs showed the same qualitative differential gene expression (corrected *p*-values < 0.05; FDR) in one or two GSEs (Table 1). In particular, *CTNNB1*, *GNAQ*, *GSK3B*, *GSTP1*, *MAPK3*, *PPP1CC*, *PRKACA*, and *SMAD4* genes (corrected *p-*value < 0.05; FDR) were over- or sub-expressed in at least two datasets of melanoma metastases when compared with primary tumor samples. *PTPN11* or protein tyrosine phosphatase, non-receptor type 11 showed upregulation (corrected *p-*value < 0.05; FDR) in the three re-analyzed independent cohorts of patients (Table 1).

## 4. Discussion

In this study, we constructed an in silico model (interactome) resulting from the interplay between stem cell pluripotency and Ca^2+^ signaling pathways, in a network composed of 294 genes/proteins and 2995 interactions based on “Experiments” and “Databases” and a confidence score of at least 0.400 (Figure 2). The newly-developed model was then subjected to further analysis by the GALANT software, showing a similar pattern of differences when the relative expression of metastatic melanoma from three microarray datasets was plotted over the expression of primary tumor samples in the “STEMCa” interactome (Figure 3). Differential expression analysis of “STEMCa” model showed significant transcriptional differences in metastatic vs. primary tumor tissues with around 51% (151 out of 294) exhibiting changes in at least one of the studied datasets (Supplementary Table S2). In particular, *CTNNB1*, *GNAQ*, *GSK3B*, *GSTP1*, *MAPK3*, *PPP1CC*, *PRKACA*, *SMAD4*, and *PTPN11* were de-regulated (corrected *p*-values < 0.05) central genes in the tissue samples in our model (Table 1 and Figure 4), representing not only a putative group of biomarkers but also potential therapeutic targets in melanoma metastasis. The observed transcriptional changes may account for the molecular changes involved in the metastatic transformation of primary tumor sites.

Melanoma is the most lethal type of skin cancer with an estimated 73,870 new cases in 2015, representing 4.5% of all new cancer cases. In this same year, 9940 people died from this disease. The incidence of melanoma is rising [25]. Metastasis is the spread of cancer cells from the primary tumor site to distant organs and accounts for more than 90% of deaths in cancer patients since most metastases are undetectable by clinicians [3,26]. Therefore, a better comprehension of the mechanisms governing melanoma metastasis and the discovery of optimal biomarkers for early diagnosis remain necessary.

In general, a couple of hallmarks make CSCs putative candidates responsible for cancer metastasis: first, it seems that these are the only cells within tumors that are capable of initiating and maintaining cancer growth; and second, a single CSC is able to develop a metastatic lesion in contrast to the rest of the cancer cells from the heterogeneous tumor [27,28]. In addition, the expression of pluripotency markers in CSCs, which share several molecular characteristics typically observed in normal SCs and depend on common embryonic signaling pathways, has been reported. These molecular pathways likely represent novel therapeutic targets that could improve patient survival rates [29].

The homeostasis of Ca^2+^ and Ca^2+^-dependent signaling are critical components of cellular fate [30]. Surprisingly, this event has not been studied in detail yet as in pluripotent SCs. The role of Ca^2+^ signaling is known in both proliferation and differentiation of SCs at the very early stages of development and, consequently, the fate of their physiopathological status in vivo [31]. Therefore, one could speculate that transcriptional differences in stem cell pluripotency and Ca^2+^ signaling-related genes may contribute to the metastatic transformation in melanoma malignancy. The results that we report here, derived from our newly-developed in silico model (Figure 2 and Supplementary Table S1), corroborate this hypothesis in three independent datasets of microarray samples (Figure 3 and Supplementary Table S2). However, the absence of experiments confirming the up or downregulation of these genes at the protein level in additional human metastatic melanoma tissues remains a limitation that requires further study.

Considering that diseases treated in the early stages are associated with better patient outcomes, the discovery of new melanoma biomarkers through OMICS (genomics, proteomics, metabolomics) and systems biology tools are required to assist clinicians in the diagnosis, follow-up, treatment, and even prediction of melanoma outcomes [32]. The use of PPI offers the opportunity to analyze the functional relationships among biological molecules within one or numerous signaling pathways [16]. Researchers can elucidate centrality values as a network-based approach to objectively identify/select candidate biomarkers and therapeutic targets in silico in the context of any disease [16,33,34] and check alterations at the transcriptional level in independent array datasets. In this study, we identified a group of central members including *CTNNB1*, *GNAQ*, *GSK3B*, *GSTP1*, *MAPK3*, *PPP1CC*, *PRKACA*, *SMAD4*, and *PTPN11* genes within the present in silico “STEMCa” interatomic model (Figure 4). Thereafter, we qualitatively validated in at least two independent datasets of patients from GEO significant differences in expression of these genes (corrected *p-*value < 0.05; FDR) (Table 1). From those, only *PTPN11* (NH-B) gene confirmed its upregulation in another third independent cohort of patients. This could be due to the lower number of samples provided in the third re-analyzed dataset with 58 samples (GSE15605), when compared with the other two GSEs with 83 and 104 samples, respectively. This group of nine genes/proteins as central (NH-Bs and HBs) de-regulated nodes occupying vulnerable points in the “STEMCa” interactome represent potential therapeutic targets for melanoma metastasis in silico and invites further investigation in vitro and in vivo.

*PTPN11* gene encodes the protein tyrosine phosphatase non-receptor type 11 and has a subnetwork contribution to the “STEMCa” interactome from the stem cell pluripotency signaling pathway (map04550) (Supplementary Table S1). It is remarkable that suppression of *PTPN11* confers sensitivity to BRAF inhibitors (used in cancer therapeutics) in colon cancer by blocking signaling from the receptor tyrosine kinases to the RAS–MEK–ERK pathway. This suppression also prevents the acquired resistance to targeted cancer drugs resulting from receptor tyrosine kinase activation [35]. According to the CSC theory, one of the main characteristics of these cells is to confer drug-resistant recurrence [1,2]. These recent findings [35] are therefore consistent with the present network-based identification of biomarkers and potential therapeutic targets from stem cell pluripotency and Ca^2+^ signaling pathways in metastatic melanomas.

We conclude that (i) altered expression of stem cell pluripotency and Ca^2+^ signaling pathway-related genes may contribute to metastatic transformation, and (ii) *CTNNB1*, *GNAQ*, *GSK3B*, *GSTP1*, *MAPK3*, *PPP1CC*, *PRKACA*, *SMAD4*, and especially *PTPN11* may represent an optimal candidate group of biomarkers and in silico therapeutic targets for melanoma metastasis.

## Figures and Tables

**Table 1:** Centrality values (*degree* and *betweenness*) for each node (gene/protein) within the stem cell pluripotency (map04550) and calcium signaling (map04020) interaction network model (“STEMCa” network). Differentially expressed genes within these members are listed. Centralities over the thresholds with values above one standard deviation of the mean (+1 SD) are in bold. Corrected *p*-values < 0.05 are considered significant for differentially expressed genes. Nodes identified as nonhub-bottlenecks (NH-Bs) and hub-bottlenecks (HBs) (based on *degree* and *betweenness* centralities), with values above +1 SD of the mean are represented as NH-Bs and HBs, respectively. “ST”, “Ca”, and “CON” represent “stem cell pluripotency”, “calcium signaling”, and “connector”, respectively. NH-Bs and HBs differentially expressed in at least two analyzed GSEs are in gray color. Only one gene (*PTPN11*, NH-B) was differentially expressed (upregulated) in the three analyzed GSEs. Table sorted by alphabetical order of gene symbol.

Gene Information			Topology		NH-B or HB	GSE8401		GSE46517		GSE15605	
Gene Symbol	Ensemble ID	Sub.	Degree	Betweenness		Diff. Exp.	Corrected *p-*Value (FDR)	Diff. Exp.	Corrected *p-*Value (FDR)	Diff. Exp.	Corrected *p-*Value (FDR)
*AKT1*	ENSP00000270202	ST	32	**0.03034247**	NH-B	---	0.073094859	Down	**0.002572769**	---	0.99159187
*CALM1*	ENSP00000349467	CON	**46**	**0.04297522**	HB	---	0.420640218	Up	**0.014354422**	---	0.102753445
*CTNNB1*	ENSP00000344456	ST	**49**	**0.09934346**	HB	Up	**0.000112883**	Up	**5.22113E−05**	---	0.134736908
*EGFR*	ENSP00000275493	Ca	**41**	**0.02867496**	HB	---	0.454469806	Down	**4.09354E−18**	---	0.372953188
*ESR1*	ENSP00000206249	CON	37	**0.03902762**	NH-B	---	0.34361801	---	0.691913409	---	0.858356832
*GABRR1*	ENSP00000412673	CON	4	**0.02033756**	NH-B	---	0.516750581	---	0.178807962	Up	**0.030236148**
*GNAQ*	ENSP00000286548	Ca	**69**	**0.08508797**	HB	Up	**0.014769785**	Up	**0.038122837**	---	0.174777485
*GSK3B*	ENSP00000324806	ST	31	**0.03985954**	NH-B	Down	**4.43231E−06**	Down	**2.75596E−10**	---	0.542667881
*GSTP1*	ENSP00000381607	CON	4	**0.02033756**	NH-B	Down	**3.14081E−05**	Down	**2.61693E−06**	---	0.274434846
*HDAC1*	ENSP00000362649	CON	27	**0.03262084**	NH-B	---	0.213434449	---	0.710999269	---	0.865172009
*IGF1*	ENSP00000302665	CON	28	**0.04312205**	NH-B	---	0.697266013	---	0.431465367	---	0.66956775
*ITPR3*	ENSP00000363435	Ca	22	**0.01942364**	NH-B	---	0.269623007	---	0.671875922	---	0.742562621
*JUN*	ENSP00000360266	CON	31	**0.03792962**	NH-B	Down	**8.92807E−06**	---	0.171366276	---	0.900656682
*MAPK1*	ENSP00000215832	ST	**42**	**0.05561423**	HB	Up	**0.000412172**	---	0.429091242	---	0.608460569
*MAPK14*	ENSP00000229794	ST	34	**0.02912488**	NH-B	---	0.605738821	Up	**0.038642425**	---	0.358766287
*MAPK3*	ENSP00000263025	ST	33	**0.02244545**	NH-B	Down	**1.10052E−05**	Down	**0.01074679**	---	0.999319565
*MYC*	ENSP00000367207	ST	34	**0.03598441**	NH-B	---	0.602836201	---	0.274057104	---	0.449615647
*NCOR1*	ENSP00000268712	CON	21	**0.02875081**	NH-B	---	0.627351808	---	0.257849878	---	0.148831132
*PIK3R1*	ENSP00000274335	ST	**69**	**0.05441236**	HB	---	0.383906559	Down	**0.030059149**	---	0.057825554
*PPP1CC*	ENSP00000335084	CON	22	**0.01965509**	NH-B	Up	**1.52233E−05**	Up	**3.22551E−05**	---	0.307997432
*PRKACA*	ENSP00000309591	CON	**51**	**0.0837037**	HB	Down	**0.00193898**	Down	**0.006166758**	---	0.056947292
*PRKCA*	ENSP00000408695	Ca	**55**	**0.03906664**	HB	Up	**0.003860994**	---	0.227826787	---	0.934220679
*PTPN11*	ENSP00000340944	ST	34	**0.02374219**	NH-B	Up	**0.039908343**	Up	**0.009092992**	Up	**0.048264037**
*SMAD2*	ENSP00000262160	ST	29	**0.02336588**	NH-B	Up	**0.009466433**	---	0.924719732	---	0.23296179
*SMAD3*	ENSP00000332973	ST	28	**0.02392531**	NH-B	---	0.975647588	---	0.09794078	---	0.219862918
*SMAD4*	ENSP00000341551	ST	35	**0.03753624**	NH-B	Up	**5.56315E−06**	Up	**0.000292094**	---	0.141417242
*TCF3*	ENSP00000262965	ST	18	**0.02573384**	NH-B	---	0.803481248	---	0.217139092	---	0.260191349
											
*Mean*			20.37414	6.56E−03							
*SD*			16.80553	0.01276070							
*1 SD*			37.17968	0.01932203							

## Supplementary Materials

The following are available online at http://www.mdpi.com/2076-3271/6/1/23/s1, Supplementary Table S1. Genes/proteins belonging to the stem cell pluripotency (map04550) and calcium signaling (map04020) interaction network model (“STEMCa” network) and its gene subnetwork contributions (ST: stem cell pluripotency; Ca2+: calcium signaling; CON: connector), Supplementary Table S2. Centrality values (degree and betweenness) and differentially expressed genes from the stem cell pluripotency (map04550) and Ca^2+^ signaling (map04020) interaction network model (“STEMCa” network). Corrected *p*-values < 0.05 were considered significant. Centralities over the thresholds with value/s above one standard deviation of the mean (+1 SD) are color-marked.
