# Supplementary material for: Network-Based Identification of Altered Stem Cell Pluripotency and Calcium Signaling Pathways in Metastatic Melanoma
Source: Med Sci (Basel). 2018 Mar 8;6(1):23. doi: 10.3390/medsci6010023 (PMC5872180; doi:10.3390/medsci6010023)
Supplement: Supplementary file 1 [file medsci-06-00023-s001.zip › Supplementary Table 2. Neves de Oliveira et al. 2018.pdf]

Supplementary Table 2. Centrality values (*degree* and *betweenness*) and differentially expressed genes from the stem cell pluripotency (map04550) and Ca^{2+} signaling (map04020) interaction network model ("STEMCa" network). Corrected *p*-values < 0.05 were considered significant. Centralities over the thresholds with value/s above one standard deviation of the mean (+1 SD) are color-marked.

GENE INFORMATION			TOPOLOGY		GSE8401			GSE46517			GSE15605		
**Gene Symbol**	**Ensemble ID**	**Subnetwork**	***Degree***	***Betweenness***	**LogFC**	**Diff. Exp.**	**Corrected *p*-value** (FDR)	**LogFC**	**Diff Exp**	**Corrected *p*-value** (FDR)	**LogFC**	**Diff. Exp.**	**Corrected *p*-value** (FDR)
ACTB	ENSP00000349960	CON	20	0.01907129	-0.024108298	no	0.761449925	-0.00686	no	0.803684421	1.559675026	**Up**	**0.032652195**
ACVR1	ENSP00000263640	ST	11	0.00718543	0.493435774	**Up**	**0.002341069**	0.014872	no	0.750579897	0.259755304	no	0.395003714
ACVR2A	ENSP00000241416	ST	15	0.00119467	-0.0283413	no	0.677864084	0.017547	no	0.634267226	-0.456205804	no	0.088499856
ACVR2B	ENSP00000340361	ST	15	0.00119467	0.125032579	no	0.105642614	0.024948	no	0.4679031	0.475372468	no	0.181619371
ADCY1	ENSP00000297323	Ca	28	0.00226065	0.770309904	**Up**	**0.003237809**	0.129314	**Up**	**0.04036285**	0.250179176	no	0.8225865
ADCY2	ENSP00000342952	Ca	31	0.00377255	-0.816673844	**Down**	**0.000913162**	-0.3964	**Down**	**4.27246E-06**	-2.246747858	**Down**	**0.019316464**
ADCY3	ENSP00000260600	Ca	32	0.00459359	0.063873407	no	0.637425296	-0.04102	no	0.23974253	-0.130007519	no	0.769024733
ADCY4	ENSP00000312126	Ca	31	0.00377255	*no probe*	*no probe*	*no probe*	*no probe*	*no probe*	*no probe*	-0.963961392	**Down**	**0.047564684**
ADCY7	ENSP00000254235	Ca	32	0.01277469	-0.047589167	no	0.816569602	-0.07034	no	0.408955104	-0.210228945	no	0.601933035
ADCY8	ENSP00000286355	Ca	33	0.00446963	-0.174215996	**Down**	**0.016616289**	-0.06279	no	0.051168453	-0.335921631	no	0.247837447
ADCY9	ENSP00000294016	Ca	31	0.00377255	-0.428191131	**Down**	**0.01814938**	-0.18131	**Down**	**0.015078196**	-0.519722729	no	0.103479907
ADORA2A	ENSP00000336630	Ca	23	0.00102844	*no probe*	*no probe*	*no probe*	*no probe*	*no probe*	*no probe*	*no probe*	*no probe*	*no probe*
ADORA2B	ENSP00000304501	Ca	22	0.000576	0.091943694	no	0.677859748	-0.03734	no	0.526103972	0.552891787	no	0.443724055
ADRA1A	ENSP00000369960	Ca	**43**	0.000634	-0.052828498	no	0.319141538	-0.07484	no	0.21525575	-0.206687591	no	0.347422003
ADRA1B	ENSP00000306662	Ca	**46**	0.00127274	-0.056029792	no	0.299706925	0.029342	no	0.65009466	-0.111464079	no	0.586795269
ADRA1D	ENSP00000368766	Ca	**43**	0.000634	-0.073784386	no	0.379436234	-0.0444	no	0.377800315	-0.204841053	no	0.424114462
ADRB1	ENSP00000358301	Ca	24	0.00197701	-0.110653483	no	0.054753207	0.024246	no	0.6296024	0.675856475	no	0.416422134
ADRB2	ENSP00000305372	Ca	29	0.00258616	-0.236878921	no	0.060548662	-0.04127	no	0.185494082	-1.529736243	**Down**	**0.013142131**
ADRB3	ENSP00000343782	Ca	21	0.00000275	-0.024500254	no	0.743641898	-0.10018	no	0.053000578	-0.385328567	no	0.100719202
AGTR1	ENSP00000273430	Ca	**48**	0.00448218	-0.052678124	no	0.731325344	0.088783	no	0.223388799	0.244480193	no	0.774308308
AKAP10	ENSP00000225737	Ca	1	0	0.111766375	no	0.142786753	0.084407	no	0.052028474	0.871407694	no	0.148502956
AKT1	ENSP00000270202	ST	32	**0.03034247**	-0.245929347	no	0.073094859	-0.11825	**Down**	**0.002572769**	-0.004555422	no	0.99159187
AKT2	ENSP00000375892	ST	10	0.00172914	0.003687556	no	0.970387807	-0.05444	no	0.414077105	0.472894843	no	0.214353755
AKT3	ENSP00000263826	ST	6	0.0000974	0.53815105	**Up**	**0.000240754**	0.019771	no	0.800831597	1.946959962	**Up**	**0.004271082**
AKTIP	ENSP00000378152	ST	1	0	-0.192604401	**Down**	**0.018985222**	-0.06075	no	0.079284466	0.141515237	no	0.687592363
ALCAM	ENSP00000305988	ST	1	0	0.014306297	no	0.960870922	-0.07282	no	0.524214628	-0.034796199	no	0.961846632
ALDH1B1	ENSP00000366927	ST	1	0	0.271807519	**Up**	**0.018479944**	0.084034	no	0.108392189	0.173448261	no	0.746313451
ALDH3A1	ENSP00000225740	ST	2	0.01360512	-1.28140477	**Down**	**7.65086E-14**	-0.36927	**Down**	**1.00595E-14**	-1.666182948	**Down**	**0.012465938**
APC	ENSP00000257430	ST	13	0.00330135	0.044452604	no	0.390786804	0.07177	no	0.172913743	0.840235375	no	0.144216088
ARAF	ENSP00000366244	ST	10	0.0000934	0.102167477	no	0.134605848	-0.02064	no	0.570084586	0.506969343	no	0.190750032

ATP2A1	ENSP00000349595	Ca	2	0.0000575	-0.031433038	no	0.740942807	-0.02371	no	0.743130158	-0.254134846	no	0.218062139
ATP2A2	ENSP00000440045	Ca	3	0.000222	0.225059947	no	0.074503034	0.003918	no	0.944783973	0.223497694	no	0.415118732
AVP	ENSP00000369647	CON	**63**	0.01721403	-0.160398739	**Down**	**0.008357143**	0.039159	no	0.364066614	-0.082562994	no	0.613046328
AVPR1A	ENSP00000299178	Ca	**43**	0.000373	0.025379757	no	0.726902328	-0.07253	no	0.213416041	-0.168858597	no	0.74270437
AVPR1B	ENSP00000356094	Ca	1	0	-0.194236639	**Down**	**0.002069273**	0.017751	no	0.633988754	-0.152096423	no	0.441488605
AXIN1	ENSP00000262320	ST	21	0.0104337	-0.102241516	no	0.081145394	-0.03999	no	0.132608851	-0.343165433	no	0.225150251
AXIN2	ENSP00000302625	ST	9	0.000238	*no probe*	*no probe*	*no probe*	*no probe*	*no probe*	*no probe*	-1.339143633	**Down**	**0.01547958**
BDKRB1	ENSP00000216629	Ca	**51**	0.00208106	-0.141151364	**Down**	**0.017239378**	-0.05364	no	0.493689279	-0.364959628	no	0.322724166
BDKRB2	ENSP00000307713	Ca	**54**	0.00547469	-0.677968642	**Down**	**1.24357E-15**	-0.42007	**Down**	**1.38507E-17**	-1.056127029	**Down**	**0.045121581**
BMI1	ENSP00000365851	ST	4	0.0000358	*no probe*	*no probe*	*no probe*	*no probe*	*no probe*	*no probe*	*no probe*	*no probe*	*no probe*
BMP2	ENSP00000368104	ST	20	0.009355	-0.222590883	no	0.297764194	0.043115	no	0.59259277	-1.288334655	**Down**	**0.015889038**
BMP4	ENSP00000245451	ST	16	0.00731462	-0.310362702	**Down**	**0.003099308**	-0.47196	**Down**	**4.18036E-05**	-0.81379411	no	0.094984367
BMP6	ENSP00000283147	ST	7	0.00000425	0.123084462	no	0.287641243	0.093497	no	0.167195897	0.528506397	no	0.264263554
BMP7	ENSP00000379204	ST	11	0.000174	-0.083799725	no	0.243686624	-0.03942	no	0.462180145	0.078075942	no	0.955706107
BMPR1A	ENSP00000224764	ST	14	0.000735	0.145059004	no	0.059506879	-0.01856	no	0.827340048	0.574343394	no	0.120578819
BMPR1B	ENSP00000264568	ST	13	0.000659	0.130798092	no	0.136287556	0.069788	no	0.121814471	-1.474246454	no	0.053777794
BMPR2	ENSP00000363708	ST	14	0.000735	-0.448336832	**Down**	**0.000118576**	-0.23117	**Down**	**3.66751E-07**	0.861342009	no	0.087437272
BRAF	ENSP00000288602	ST	14	0.000595	0.186639374	**Up**	**0.00209567**	0.097903	**Up**	**0.004428605**	0.653803191	no	0.21370785
CACNA1A	ENSP00000353362	Ca	5	0.000403	-0.031591228	no	0.751838231	-0.17586	**Down**	**0.004772247**	-0.064931331	no	0.905752777
CACNA1B	ENSP00000360406	Ca	7	0	-0.24689251	**Down**	**0.001150002**	0.03364	no	0.466035081	-0.564549564	no	0.101426239
CACNA1C	ENSP00000266376	Ca	17	0.005718	-0.099400691	no	0.174228705	-0.09854	no	0.060230691	-0.41947977	no	0.374748958
CACNA1D	ENSP00000288139	Ca	16	0.00632295	-0.095407579	no	0.169386086	-0.0188	no	0.798179115	-1.19305511	no	0.086683789
CACNA1E	ENSP00000356545	Ca	7	0	-0.062648998	no	0.332426359	0.009584	no	0.862237025	-0.376419091	no	0.324810026
CACNA1F	ENSP00000365441	Ca	7	0	0.110494279	no	0.181265161	-0.05571	no	0.313058214	-0.215498325	no	0.227971683
CACNA1G	ENSP00000352011	Ca	12	0.00971323	-0.079662181	no	0.196739841	-0.03226	no	0.682084341	-0.554730986	no	0.109883555
CACNA1H	ENSP00000334198	Ca	10	0.000901	-0.147646031	**Down**	**0.018103666**	-0.04337	no	0.303674783	-0.344074896	no	0.146501819
CACNA1I	ENSP00000385019	Ca	7	0.000017	-0.030942	no	0.68026309	0.015795	no	0.715554113	-0.094909931	no	0.783393576
CACNA1S	ENSP00000355192	Ca	14	0.00431991	-0.077981502	no	0.159584796	0.049286	no	0.278558668	-0.033481743	no	0.845326922
CALM1	ENSP00000349467	CON	**46**	**0.04297522**	0.05970491	no	0.420640218	0.082097	**Up**	**0.014354422**	0.898796891	no	0.102753445
CALM2	ENSP00000272298	Ca	11	0.00310394	*no probe*	*no probe*	*no probe*	*no probe*	*no probe*	*no probe*	*no probe*	*no probe*	*no probe*
CAMK2B	ENSP00000379098	Ca	29	0.00928585	-0.017180559	no	0.696252539	-0.0208	no	0.825450834	-0.036004175	no	0.934238294
CAMK4	ENSP00000282356	Ca	7	0.000468	0.082553391	no	0.235787616	0.150469	**Up**	**0.000955151**	0.212876403	no	0.800197653
CCKAR	ENSP00000295589	Ca	**42**	0.00000382	-0.114222594	no	0.106696824	0.013983	no	0.76901408	-0.046934205	no	0.900241907
CCKBR	ENSP00000335544	Ca	**43**	0.00127413	-0.15080949	**Down**	**0.025703471**	-0.02904	no	0.368975123	-0.423549775	no	0.165528817
CCND1	ENSP00000227507	CON	18	0.0073975	-0.786599389	**Down**	**0.00159198**	-0.22917	**Down**	**0.005067749**	-0.100126092	no	0.852703841
CD38	ENSP00000226279	Ca	6	0.000342	0.633163266	**Up**	**0.00152246**	0.14165	**Up**	**0.007978865**	1.375678242	**Up**	**0.005307694**
CD44	ENSP00000398632	ST	10	0.00229193	-0.443061465	**Down**	**0.022986638**	0.131686	no	0.179109005	0.490288672	no	0.479148798

CD6	ENSP00000323280	ST	2	0.00682594	-0.407181124	**Down**	**0.002998311**	0.067237	no	0.711281452	-0.415584687	no	0.496054969
CDH5	ENSP00000344115	CON	6	0.01369088	-0.229501486	no	0.139755758	-0.2715	**Down**	**0.000198259**	-0.305482103	no	0.390122841
CHRM1	ENSP00000306490	Ca	**49**	0.00816648	*no probe*	*no probe*	*no probe*	*no probe*	*no probe*	*no probe*	-0.848163445	no	0.08631478
CHRM2	ENSP00000319984	Ca	16	0.000846	0.102543657	no	0.368022426	0.148074	**Up**	**2.72041E-05**	-0.141731168	no	0.446372646
CHRM3	ENSP00000255380	Ca	**44**	0.000224	0.302635974	**Up**	**0.033554266**	0.048657	no	0.704420478	-0.257621355	no	0.776247495
CHRM5	ENSP00000372750	Ca	**44**	0.000224	0.086640245	no	0.35743343	0.064373	no	0.098808445	-0.211711882	no	0.325431383
CHRNA7	ENSP00000407546	Ca	1	0	*no probe*	*no probe*	*no probe*	*no probe*	*no probe*	*no probe*	*no probe*	*no probe*	*no probe*
CTNNA1	ENSP00000304669	ST	16	0.00547324	-0.423084932	**Down**	**0.001525878**	-0.09298	no	0.141453598	1.150609889	no	0.060574303
CTNNA2	ENSP00000418191	ST	8	0.000443	-0.146311509	no	0.189635269	0.044375	no	0.452861071	-0.081112324	no	0.920987621
CTNNA3	ENSP00000362849	ST	3	0.0000359	-0.034442392	no	0.606649134	-0.04157	no	0.268857768	-0.177100562	no	0.422572677
CTNNB1	ENSP00000344456	ST	**49**	**0.09934346**	0.573777007	**Up**	**0.000112883**	0.161343	**Up**	**5.22113E-05**	0.801985615	no	0.134736908
CTNNBIP1	ENSP00000366466	ST	7	0.000349	-0.771669249	**Down**	**1.86249E-13**	-0.27636	**Down**	**5.36471E-16**	-0.798545079	**Down**	**0.031494238**
CTNND2	ENSP00000307134	ST	2	0.000215	-0.175894329	**Down**	**0.000467185**	0.03196	no	0.594139458	-0.568506595	no	0.275886549
CYSLTR1	ENSP00000362401	Ca	**42**	0.00000382	-0.066353691	no	0.059370283	0.042701	no	0.525517399	-0.359284529	no	0.425823745
CYSLTR2	ENSP00000282018	Ca	**42**	0.00000382	0.006319143	no	0.961072995	-0.01204	no	0.873988316	-0.645886751	no	0.233398296
DLX5	ENSP00000222598	ST	1	0	-0.268598333	**Down**	**3.33381E-05**	-0.01215	no	0.728025125	0.110247434	no	0.840731238
DRD1	ENSP00000327652	Ca	23	0.001007	0.013983884	no	0.787069697	0.102939	**Up**	**0.016480638**	-0.19250745	no	0.242391909
DRD5	ENSP00000306129	Ca	22	0.000576	0.055393592	no	0.592811441	0.005495	no	0.868778465	-0.01086879	no	0.946459053
DVL1	ENSP00000368169	ST	24	0.01655107	-0.251755594	**Down**	**0.02991641**	-0.04217	no	0.423828376	-0.879342464	**Down**	**0.004058239**
EDNRA	ENSP00000315011	Ca	**54**	0.00594781	-0.152646475	**Down**	**0.00389713**	-0.23916	**Down**	**0.000373873**	-0.463940959	no	0.454813356
EDNRB	ENSP00000366416	Ca	**45**	0.00161893	0.06962535	no	0.867511022	-0.03943	no	0.802086629	-0.165741295	no	0.788352776
EGFR	ENSP00000275493	Ca	**41**	**0.02867496**	-0.074739452	no	0.454469806	-0.77396	**Down**	**4.09354E-18**	-0.91200768	no	0.372953188
ERBB2	ENSP00000269571	Ca	23	0.00599284	-0.771670613	**Down**	**4.23948E-10**	-0.29477	**Down**	**1.84539E-10**	-0.579780806	no	0.171349238
ERBB3	ENSP00000267101	Ca	14	0.00208024	0.016327019	no	0.968562333	-0.04873	no	0.682084341	-0.016214834	no	0.984742649
ERBB4	ENSP00000342235	Ca	18	0.00462692	0.145840034	no	0.060516277	-0.06295	no	0.122525084	0.129515459	no	0.898711344
ESR1	ENSP00000206249	CON	37	**0.03902762**	0.065573168	no	0.34361801	-0.03276	no	0.691913409	-0.121373719	no	0.858356832
ETV4	ENSP00000321835	ST	1	0	0.046482663	no	0.791958206	0.029367	no	0.79411606	1.036812913	**Up**	**0.029115616**
ETV5	ENSP00000306894	ST	1	0	0.260189791	no	0.33458146	-0.14935	no	0.068682414	1.201575161	no	0.081140066
F2R	ENSP00000321326	Ca	**48**	0.01178359	0.602243494	**Up**	**0.023147566**	0.108833	no	0.176177758	-0.534595111	no	0.346116148
FGF2	ENSP00000264498	ST	14	0.00281367	0.106923208	no	0.101486883	-0.006	no	0.946133832	0.230020218	no	0.692780856
FGFR1	ENSP00000393312	ST	16	0.00132134	-0.169012033	no	0.385036119	-0.17179	no	0.059545658	0.021948761	no	0.973762337
FGFR2	ENSP00000410294	ST	16	0.00237305	-0.043788263	no	0.54666231	-0.49196	**Down**	**2.8766E-13**	-2.893741608	**Down**	**0.006270883**
FGFR3	ENSP00000339824	ST	10	0.000465	-2.51545166	**Down**	**4.2596E-13**	-1.11192	**Down**	**7.5847E-19**	-3.214335236	**Down**	**0.006124764**
FGFR4	ENSP00000292408	ST	9	0.000477	0.084377835	no	0.230262848	0.147837	**Up**	**0.026484362**	-0.273961519	no	0.390185329
FZD1	ENSP00000287934	ST	25	0.00690974	0.26987118	no	0.255895831	0.040916	no	0.694950224	0.648658056	no	0.0820422
FZD10	ENSP00000229030	ST	21	0.00437685	-0.715983427	**Down**	**2.49109E-11**	-0.32664	**Down**	**3.50974E-15**	-1.526629405	**Down**	**0.023253115**
FZD2	ENSP00000323901	ST	28	0.00702029	-0.088095883	no	0.410641519	-0.00588	no	0.920622314	0.559371415	no	0.081095083
FZD3	ENSP00000240093	ST	25	0.00437685	0.243648857	**Up**	**0.007698645**	0.132634	**Up**	**0.000386567**	0.600345554	no	0.184691037

FZD4	ENSP00000434034	ST	27	0.00438291	-0.035353176	no	0.70435913	-0.04727	no	0.075469187	-0.403310645	no	0.245860687
FZD5	ENSP00000354607	ST	31	0.0172332	-0.072368926	no	0.291494394	0.064076	no	0.14874996	0.394866852	no	0.355591884
FZD6	ENSP00000351605	ST	25	0.00437685	0.476450171	no	0.075135475	-0.01869	no	0.839819858	-0.500216734	no	0.254136444
FZD7	ENSP00000286201	ST	25	0.00437986	0.42773233	no	0.080845101	0.05132	no	0.647617036	-0.180184209	no	0.750111855
FZD8	ENSP00000363826	ST	25	0.0063776	-0.040342841	no	0.67344977	0.001689	no	0.977696363	0.184641108	no	0.814632786
FZD9	ENSP00000345785	ST	3	0	-0.065039336	no	0.582377552	0.056973	no	0.363924495	0.019023728	no	0.966747866
GABRR1	ENSP00000412673	CON	4	**0.02033756**	0.043553532	no	0.516750581	0.045095	no	0.178807962	0.970277925	**Up**	**0.030236148**
GDF5	ENSP00000363489	ST	9	0.0000148	0.008037279	no	0.923245213	0.017108	no	0.800684561	-0.100366752	no	0.590368797
GNA11	ENSP00000078429	Ca	**52**	0.00607334	-0.139746345	no	0.247003933	0.412342	**Up**	**0.000111027**	0.041139788	no	0.929693492
GNA14	ENSP00000365807	Ca	**52**	0.00607334	0.087397394	no	0.235043887	-0.05462	no	0.321151731	0.175716186	no	0.746873279
GNA15	ENSP00000262958	Ca	**55**	0.0073464	-1.283552441	**Down**	**1.41761E-13**	-0.56968	**Down**	**1.24793E-15**	-1.227252208	**Down**	**0.042581416**
GNAL	ENSP00000334051	Ca	28	0.00363512	-0.611900981	**Down**	**0.003427336**	-0.69432	**Down**	**6.07879E-09**	-1.080828356	no	0.131120178
GNAQ	ENSP00000286548	Ca	**69**	**0.08508797**	0.298487114	**Up**	**0.014769785**	0.073427	**Up**	**0.038122837**	0.74299322	no	0.174777485
GNAS	ENSP00000360141	Ca	**40**	0.01161783	0.116809053	no	0.297398232	-0.08881	no	0.154063105	0.496928684	no	0.351157727
GRB2	ENSP00000339007	ST	34	0.01239059	0.1341608	no	0.173913017	0.034527	no	0.407235369	0.681520821	no	0.136051561
GRIN1	ENSP00000360608	Ca	18	0.00267627	-0.024483637	no	0.784253651	-0.07325	no	0.401534161	-0.200395883	no	0.362422113
GRIN2A	ENSP00000332549	Ca	12	0.000185	-0.019978253	no	0.826241727	0.023598	no	0.658449765	-1.098856784	**Down**	**0.040289652**
GRIN2C	ENSP00000293190	Ca	10	0.0000828	-0.067607325	no	0.272545164	0.000465	no	0.993925086	-0.030658879	no	0.888447139
GRIN2D	ENSP00000263269	Ca	10	0.0000828	0.017640261	no	0.818721006	-0.0684	no	0.231573836	-0.15393082	no	0.810190058
GRM1	ENSP00000282753	Ca	**46**	0.00168435	0.023891092	no	0.670940189	-0.23128	**Down**	**0.007888872**	-0.52683163	no	0.295472353
GRM5	ENSP00000306138	Ca	**48**	0.0037303	-0.090157207	no	0.100362644	0.117849	**Up**	**0.034460137**	-0.290592791	no	0.345508814
GRPR	ENSP00000369643	Ca	**42**	0.00000382	-0.055544305	no	0.474088705	-0.08311	no	0.090456741	0.045101347	no	0.835932347
GSK3B	ENSP00000324806	ST	31	**0.03985954**	-0.562875324	**Down**	**4.43231E-06**	-0.18247	**Down**	**2.75596E-10**	-0.201008769	no	0.542667881
GSTP1	ENSP00000381607	CON	4	**0.02033756**	-0.812350991	**Down**	**3.14081E-05**	-0.23641	**Down**	**2.61693E-06**	0.543942919	no	0.274434846
HAND1	ENSP00000231121	ST	3	0.0000725	0.079389499	no	0.347372449	0.042779	no	0.174536039	-0.265579967	no	0.238294632
HDAC1	ENSP00000362649	CON	27	**0.03262084**	0.173355672	no	0.213434449	0.019098	no	0.710999269	0.038203525	no	0.865172009
HESX1	ENSP00000295934	ST	1	0	0.04694851	no	0.36173578	0.060553	no	0.335074905	0.521908034	no	0.218895253
HOXB1	ENSP00000355140	ST	2	0.00682594	-0.17990224	**Down**	**0.007911256**	-0.0026	no	0.970308301	-0.22483583	no	0.233983031
HRAS	ENSP00000309845	ST	37	0.01049836	-0.890992933	**Down**	**2.55324E-09**	-0.32771	**Down**	**2.43558E-10**	-0.711097808	no	0.077884419
HRH1	ENSP00000380247	Ca	**43**	0.00047	-0.075554575	no	0.374269443	0.012249	no	0.827340048	-0.657358695	no	0.226640155
HRH2	ENSP00000366506	Ca	21	0.00000275	-0.127908575	no	0.101410648	-0.01709	no	0.6830089	-0.186779109	no	0.365605641
HTR2A	ENSP00000367959	Ca	**45**	0.00426483	-0.05947057	no	0.302758145	0.130321	**Up**	**0.004314674**	0.039039577	no	0.954111957
HTR4	ENSP00000353915	Ca	21	0.00000275	-0.17415617	**Down**	**0.005873678**	-0.07907	no	0.081920506	-0.182156995	no	0.347566203
HTR6	ENSP00000289753	Ca	22	0.000576	-0.07268614	no	0.22694529	0.027427	no	0.615494184	-0.111423257	no	0.631752012
HTR7	ENSP00000337949	Ca	21	0.00000275	-0.121433044	**Down**	**0.025065647**	0.138847	**Up**	**0.031840716**	-0.493412135	no	0.093203046
ID1	ENSP00000365280	ST	3	0.000171	-1.20657512	**Down**	**5.70183E-08**	-0.34618	**Down**	**1.86906E-12**	-0.946741614	no	0.166337906
ID2	ENSP00000234091	ST	4	0.00116652	-0.566939734	**Down**	**0.002398096**	-0.21576	**Down**	**0.000386567**	0.625448485	no	0.262500108
ID3	ENSP00000363689	ST	2	0.00682594	-0.298897543	no	0.163554189	-0.197	**Down**	**0.008576684**	0.259162096	no	0.626432588
ID4	ENSP00000367972	ST	1	0	-0.591551548	**Down**	**0.014200785**	-0.19902	**Down**	**0.013756553**	-0.349630164	no	0.611394445

IGF1	ENSP00000302665	CON	28	**0.04312205**	-0.051563378	no	0.697266013	-0.04749	no	0.431465367	0.280476932	no	0.66956775
IGFBP1	ENSP00000275525	ST	2	0	0.014837928	no	0.683260678	0.047583	no	0.147149277	-0.060233499	no	0.835202534
IGFBP2	ENSP00000233809	ST	1	0	0.039337636	no	0.918994593	0.02773	no	0.849651004	0.958854082	no	0.228519244
IGFBP3	ENSP00000370473	ST	4	0.000407	0.134586986	no	0.689410653	0.033758	no	0.74530573	2.012582224	**Up**	**0.002001928**
IGFBP4	ENSP00000269593	ST	4	0.00227551	-0.639249613	**Down**	**0.000777461**	-0.29511	**Down**	**6.12729E-05**	-0.013719181	no	0.98024487
IGFBP5	ENSP00000233813	ST	2	0	-0.408632308	no	0.125914093	-0.17395	no	0.054306871	0.907159497	no	0.246507093
IGFBP6	ENSP00000301464	ST	1	0	-0.135127596	no	0.498251851	-0.04472	no	0.473019	-0.199420337	no	0.743044626
IGFBP7	ENSP00000295666	ST	3	0.000369	-0.410362123	no	0.072930625	-0.24646	**Down**	**0.002606339**	0.202352782	no	0.77658848
ISL1	ENSP00000230658	ST	1	0	0.102795663	no	0.108009652	0.11512	**Up**	**0.005415467**	-0.631655977	no	0.423925135
ITPR1	ENSP00000306253	Ca	23	0.01510186	0.303120385	**Up**	**0.045119675**	0.339227	**Up**	**0.002108493**	0.928933616	no	0.119391173
ITPR2	ENSP00000370744	Ca	18	0.00867089	-0.065066639	no	0.370178882	-0.18733	**Down**	**0.000384716**	-0.19287095	no	0.710105482
ITPR3	ENSP00000363435	Ca	22	**0.01942364**	-0.178803357	no	0.269623007	0.030351	no	0.671875922	0.167911281	no	0.742562621
JARID2	ENSP00000341280	ST	1	0	-0.096639831	no	0.451835097	-0.00431	no	0.940304344	-0.041136497	no	0.933851652
JUN	ENSP00000360266	CON	31	**0.03792962**	-0.735200966	**Down**	**8.92807E-06**	-0.12043	no	0.171366276	-0.060977387	no	0.900656682
KAT2B	ENSP00000263754	CON	18	0.00631839	0.322678008	no	0.253473925	0.044638	no	0.649524223	0.140351516	no	0.729777227
KAT6A	ENSP00000265713	ST	3	0.0000055	0.080286036	no	0.406750049	-0.04508	no	0.427032116	0.734824535	no	0.137127411
KIT	ENSP00000288135	ST	20	0.00142449	-1.50525054	**Down**	**8.62263E-05**	-0.625	**Down**	**8.21928E-07**	-1.867717131	**Down**	**0.016638631**
KLF4	ENSP00000363804	ST	8	0.00119229	-2.194965499	**Down**	**1.09919E-15**	-0.63434	**Down**	**3.76663E-16**	-1.72802665	**Down**	**0.001527922**
LEF1	ENSP00000265165	ST	13	0.00274525	0.269309477	no	0.283553227	0.054432	no	0.511938812	1.08833862	no	0.059588598
LEFTY2	ENSP00000355785	ST	2	0.000352	0.003992401	no	0.948810225	0.032553	no	0.549652823	-0.013158874	no	0.977199848
LHCGR	ENSP00000294954	Ca	24	0.000775	0.017386812	no	0.654791523	0.090285	no	0.081237611	-0.002077043	no	0.993490415
LIF	ENSP00000249075	ST	4	0.00031	0.445287786	**Up**	**0.014539159**	0.341029	**Up**	**0.002798926**	0.406161416	no	0.581372306
LIFR	ENSP00000263409	ST	4	0.000277	0.039094127	no	0.405779435	0.038375	no	0.488361884	0.673792536	no	0.386880495
LTB4R2	ENSP00000433290	Ca	**43**	0.00168384	-0.445011631	**Down**	**1.87791E-05**	-0.19905	**Down**	**4.19141E-05**	-0.37115823	no	0.120465774
LYN	ENSP00000428924	ST	26	0.00509551	0.130562316	no	0.671440616	0.094922	no	0.275706268	0.808851305	no	0.131719757
MAOA	ENSP00000340684	CON	2	0.00682594	-0.395192052	**Down**	**0.000230297**	-0.31348	**Down**	**2.39977E-05**	-0.756223586	no	0.308784189
MAP3K1	ENSP00000382423	ST	17	0.00469399	0.045276909	no	0.688475865	-0.06462	no	0.204972277	0.973001685	**Up**	**0.009713086**
MAPK1	ENSP00000215832	ST	**42**	**0.05561423**	0.591740009	**Up**	**0.000412172**	0.039168	no	0.429091242	-0.21478692	no	0.608460569
MAPK11	ENSP00000333685	ST	17	0.00162874	-0.130624401	**Down**	**0.015992669**	0.014487	no	0.905173664	-0.719871659	no	0.055827264
MAPK12	ENSP00000215659	ST	14	0.000758	0.074702443	no	0.643513785	0.006123	no	0.933388371	0.379637666	no	0.306037969
MAPK13	ENSP00000211287	ST	8	0.00000825	-0.795184994	**Down**	**7.35263E-10**	-0.44636	**Down**	**4.3647E-12**	-1.276705319	no	0.097217754
MAPK14	ENSP00000229794	ST	34	**0.02912488**	0.054819658	no	0.605738821	0.116615	**Up**	**0.038642425**	0.336784279	no	0.358766287
MAPK3	ENSP00000263025	ST	33	**0.02244545**	-0.299813742	**Down**	**1.10052E-05**	-0.08753	**Down**	**0.01074679**	-0.000265318	no	0.999319565
MEIS1	ENSP00000272369	ST	1	0	-0.0003252	no	0.997341657	0.015894	no	0.682437074	0.195521186	no	0.668221819
MSC	ENSP00000321445	ST	2	0	-0.44184409	**Down**	**0.0021032**	-0.31033	**Down**	**5.89108E-05**	-0.980688618	no	0.090754547
MYC	ENSP00000367207	ST	34	**0.03598441**	-0.132654894	no	0.602836201	-0.087	no	0.274057104	0.408910137	no	0.449615647
MYLK	ENSP00000353452	Ca	6	0.0000433	-0.579985987	**Down**	**0.002900157**	-0.18444	**Down**	**0.003766651**	0.782758251	no	0.237182346
NANOG	ENSP00000229307	ST	9	0.000723	-0.007551062	no	0.917402394	0.05734	no	0.433134813	-0.524979813	no	0.325431383
NANOGP1	ENSP00000432545	ST	1	0	*no probe*	*no probe*	*no probe*	*no probe*	*no probe*	*no probe*	*no probe*	*no probe*	*no probe*

NCOR1	ENSP00000268712	CON	21	**0.02875081**	-0.059889718	no	0.627351808	-0.03562	no	0.257849878	0.460598803	no	0.148831132
NEUROG1	ENSP00000317580	ST	2	0	-0.145916242	**Down**	**0.010671931**	-0.10119	**Down**	**0.009517992**	-0.11084719	no	0.451717158
NHP2	ENSP00000274606	CON	2	0.0011897	-0.076409985	no	0.511599706	-0.03239	no	0.347469468	0.00238762	no	0.99490292
NODAL	ENSP00000287139	ST	6	0.00104798	-0.048888979	no	0.602381853	0.11513	**Up**	**0.023979605**	-0.131892887	no	0.569811133
NOS1	ENSP00000337459	Ca	32	0.01553999	-0.077658425	no	0.156559391	-0.11754	no	0.14979999	-0.701285144	no	0.122362533
NOS2	ENSP00000327251	Ca	8	0.000749	-0.148961943	**Down**	**0.015913971**	-0.08347	**Down**	**0.007454467**	-0.192245648	no	0.414433232
NOS3	ENSP00000297494	Ca	18	0.00431314	0.129624134	no	0.171999398	0.103497	**Up**	**0.007516629**	0.043514375	no	0.883564244
NRAS	ENSP00000358548	ST	27	0.00308327	0.802284309	**Up**	**4.36087E-06**	0.230293	**Up**	**0.000147968**	0.103512882	no	0.754482623
NTSR1	ENSP00000359532	Ca	**42**	0.00000382	-0.012939618	no	0.845700513	-0.04978	no	0.489484169	-0.459394248	no	0.359347726
ONECUT1	ENSP00000302630	ST	2	0	0.003745174	no	0.962306229	0.00058	no	0.99577601	-0.127167075	no	0.481234016
ORAI1	ENSP00000328216	Ca	6	0.00675581	*no probe*	*no probe*	*no probe*	*no probe*	*no probe*	*no probe*	-0.209950928	no	0.573666687
ORAI2	ENSP00000348752	Ca	2	0	0.061985934	no	0.397323384	0.038541	no	0.612493991	1.418784624	no	0.085557761
ORAI3	ENSP00000322249	Ca	3	0	0.041622657	no	0.708847963	-0.02418	no	0.490904569	0.339134672	no	0.314546818
OTX1	ENSP00000282549	ST	1	0	*no probe*	*no probe*	*no probe*	*no probe*	*no probe*	*no probe*	-0.177142623	no	0.235525365
OXTR	ENSP00000324270	Ca	**46**	0.00449222	-0.00710418	no	0.929951657	0.007858	no	0.822896304	0.244700775	no	0.492795142
P2RX1	ENSP00000225538	Ca	1	0	-0.066747191	no	0.36165847	0.05828	no	0.082160849	-0.1585924	no	0.6512679
P2RX2	ENSP00000343339	Ca	3	0.0136285	-0.069609197	no	0.294649184	-0.00956	no	0.818396972	-0.433467644	**Down**	**0.042320371**
P2RX3	ENSP00000263314	Ca	1	0	-0.016075485	no	0.766270034	0.097248	no	0.057139593	-0.03548392	no	0.841640553
P2RX4	ENSP00000336607	Ca	1	0	0.412726095	**Up**	**0.005131297**	0.096306	**Up**	**0.00916174**	0.23700254	no	0.609447321
P2RX6	ENSP00000416193	Ca	1	0	-0.114025437	no	0.080183886	0.145606	**Up**	**0.000205534**	-0.0778463	no	0.721910798
P2RX7	ENSP00000442349	Ca	1	0	-0.556898902	**Down**	**0.009570002**	-0.08705	no	0.077134747	-0.013761729	no	0.985812605
PAX6	ENSP00000368401	ST	7	0.01430283	-0.023637145	no	0.815807945	-0.04449	no	0.555753933	-0.123458766	no	0.832131827
PDGFRA	ENSP00000257290	Ca	19	0.00114609	-0.799235614	**Down**	**0.000702045**	-0.02633	no	0.629107468	0.688206632	no	0.255263685
PDGFRB	ENSP00000261799	Ca	24	0.00281377	-0.572694082	**Down**	**9.96889E-05**	-0.32633	**Down**	**1.79765E-06**	0.066832333	no	0.898623877
PHKG1	ENSP00000297373	Ca	2	0	-0.08593137	no	0.210806349	0.05123	no	0.146889631	-0.080881259	no	0.639821948
PIK3CG	ENSP00000352121	ST	35	0.0053544	0.131659975	no	0.127934481	-0.11271	no	0.211988872	0.323612116	no	0.62727963
PIK3R1	ENSP00000274335	ST	**69**	**0.05441236**	-0.176014314	no	0.383906559	-0.12078	**Down**	**0.030059149**	0.834032706	no	0.057825554
PIK3R5	ENSP00000392812	ST	33	0.00494904	-0.025525624	no	0.719392022	-0.00378	no	0.901719996	-0.813811885	no	0.10381709
PLCB1	ENSP00000338185	Ca	**68**	0.01829564	0.087306204	no	0.337552618	0.109468	no	0.159596073	-0.492895971	no	0.444877184
PLCB2	ENSP00000260402	Ca	**66**	0.01380784	-0.047274128	no	0.509178203	-0.09286	no	0.109728175	-0.060798796	no	0.893980024
PLCB3	ENSP00000279230	Ca	**66**	0.01380784	-0.333402195	**Down**	**2.05604E-05**	-0.00961	no	0.850449402	-0.481368338	**Down**	**0.02030294**
PLCB4	ENSP00000334105	Ca	**66**	0.01320147	0.109592421	no	0.384080228	0.079701	no	0.452082054	0.123290515	no	0.92354817
PLCD1	ENSP00000430344	Ca	18	0.000326	-0.320143416	**Down**	**8.46033E-08**	-0.06417	no	0.169646016	-0.635162508	no	0.108759044
PLCD3	ENSP00000313731	Ca	17	0.000153	*no probe*	*no probe*	*no probe*	*no probe*	*no probe*	*no probe*	-0.612677715	**Down**	**0.004968125**
PLCD4	ENSP00000388631	Ca	17	0.000153	*no probe*	*no probe*	*no probe*	*no probe*	*no probe*	*no probe*	-1.151376888	**Down**	**0.016222404**
PLCE1	ENSP00000260766	Ca	21	0.000526	0.004084141	no	0.958541755	0.041715	no	0.475458787	0.898823901	no	0.178537484

PLCG1	ENSP00000244007	Ca	**49**	0.01645968	0.034969043	no	0.747405232	0.036399	no	0.38789858	0.555452063	**Up**	**0.024680198**
PLN	ENSP00000350132	Ca	4	0.00144688	-0.047958773	no	0.74195927	0.02075	no	0.801210628	0.795372779	no	0.295586562
POU5F1	ENSP00000259915	ST	7	0.00066	*no probe*	*no probe*	*no probe*	*no probe*	*no probe*	*no probe*	*no probe*	*no probe*	*no probe*
PPIF	ENSP00000225174	Ca	6	0.01127904	0.191873255	no	0.198210866	0.082622	no	0.213416041	0.731576058	no	0.197484379
PPP1CC	ENSP00000335084	CON	22	**0.01965509**	0.609747304	**Up**	**1.52233E-05**	0.157544	**Up**	**3.22551E-05**	0.167375812	no	0.307997432
PRC1	ENSP00000377793	ST	3	0	0.605005497	**Up**	**0.004295023**	0.142992	**Up**	**0.009293472**	0.13587168	no	0.728506463
PRKACA	ENSP00000309591	CON	**51**	**0.0837037**	-0.229739375	**Down**	**0.00193898**	-0.10236	**Down**	**0.006166758**	0.577944363	no	0.056947292
PRKCA	ENSP00000408695	Ca	**55**	**0.03906664**	0.476902328	**Up**	**0.003860994**	-0.0969	no	0.227826787	-0.048769984	no	0.934220679
PRKCB	ENSP00000305355	Ca	**44**	0.0169036	0.322588256	no	0.314271066	-0.03037	no	0.600954741	0.286916522	no	0.754843222
PRKCG	ENSP00000263431	Ca	35	0.0071681	-0.111048826	no	0.102029504	-0.08034	no	0.238398963	-0.165918417	no	0.355109799
PROM1	ENSP00000415481	ST	1	0	0.060273534	no	0.315180126	0.094452	**Up**	**0.015040622**	0.262971426	no	0.78326559
PTAFR	ENSP00000301974	Ca	**43**	0.00208304	-0.011675406	no	0.925457757	-0.16196	**Down**	**0.012312197**	-0.303528499	no	0.542361345
PTEN	ENSP00000361021	ST	22	0.00613556	0.37856366	**Up**	**0.004000411**	0.074292	no	0.23554371	-0.076317161	no	0.865182847
PTGER1	ENSP00000292513	Ca	**45**	0.000485	-0.216545773	**Down**	**0.000063034**	-0.06608	no	0.476587127	-0.220067713	no	0.339085974
PTGER3	ENSP00000349003	Ca	15	0.000279	-0.01796286	no	0.794789452	0.214549	**Up**	**0.001120931**	-1.465898247	**Down**	**0.042834648**
PTGFR	ENSP00000359793	Ca	**42**	0.00000382	-0.173437897	**Down**	**0.011621634**	-0.09078	**Down**	**0.049374287**	-0.734212216	no	0.104475344
PTK2B	ENSP00000332816	Ca	20	0.00295102	-0.305563784	**Down**	**0.011750578**	-0.14236	**Down**	**0.010198577**	-0.34433873	no	0.385406587
PTPN11	ENSP00000340944	ST	34	0.02374219	0.348588259	**Up**	**0.039908343**	0.14705	**Up**	**0.009092992**	1.207936326	**Up**	**0.048264037**
RAF1	ENSP00000251849	ST	27	0.01186176	-0.122772265	no	0.248641936	-0.07943	**Down**	**0.00124894**	0.559790317	no	0.183138052
REST	ENSP00000311816	ST	2	0	-0.097581434	no	0.243497937	0.254397	**Up**	**0.000310556**	1.009594871	**Up**	**0.024198881**
RIF1	ENSP00000243326	ST	1	0	-0.003397582	no	0.942112086	0.03343	no	0.688044015	1.500650292	**Up**	**0.038049024**
RRAS	ENSP00000246792	ST	12	0.000282	-0.377633449	**Down**	**0.000603199**	-0.16525	**Down**	**5.35327E-05**	-0.482769566	no	0.289382644
RYR1	ENSP00000352608	Ca	7	0.000699	-0.469446952	**Down**	**0.01358497**	0.004308	no	0.948027757	-0.262756533	no	0.733136393
RYR2	ENSP00000355533	Ca	10	0.000959	-0.076448117	no	0.152839664	0.217964	**Up**	**0.000231604**	-0.347690769	no	0.668423945
RYR3	ENSP00000373884	Ca	4	0.0000905	0.042931871	no	0.340210558	0.016311	no	0.601497128	-1.045794583	**Down**	**0.03507429**
SALL4	ENSP00000217086	CON	10	0.00803744	*no probe*	*no probe*	*no probe*	*no probe*	*no probe*	*no probe*	0.414972234	no	0.310725094
SDC1	ENSP00000254351	CON	7	0.00959994	-1.517841904	**Down**	**3.24338E-08**	-0.88533	**Down**	**1.98433E-18**	-1.209817907	no	0.120751119
SETDB1	ENSP00000271640	ST	4	0.00687492	-0.082886696	no	0.388790347	-0.00268	no	0.956762155	0.347017789	no	0.33229295
SKIL	ENSP00000259119	ST	7	0.0000762	0.080206068	no	0.184974683	0.243431	**Up**	**0.000961394**	1.217523077	**Up**	**0.042111939**
SLC25A4	ENSP00000281456	Ca	4	0	-0.070555962	no	0.758605509	-0.03586	no	0.509293775	-0.173522016	no	0.753328025
SMAD2	ENSP00000262160	ST	29	**0.02336588**	0.210662187	**Up**	**0.009466433**	-0.00593	no	0.924719732	-0.352594394	no	0.23296179
SMAD3	ENSP00000332973	ST	28	**0.02392531**	0.004737328	no	0.975647588	0.107429	no	0.09794078	-0.389901573	no	0.219862918
SMAD4	ENSP00000341551	ST	35	**0.03753624**	0.791374313	**Up**	**5.56315E-06**	0.169068	**Up**	**0.000292094**	0.767650726	no	0.141417242
SMAD6	ENSP00000288840	ST	11	0.0000636	-0.175308617	**Down**	**0.003524164**	-0.20426	**Down**	**0.011566706**	-0.258314473	no	0.613723435
SMAD7	ENSP00000262158	ST	23	0.01209405	-0.028194174	no	0.804157049	-0.08113	no	0.091548427	0.276889098	no	0.475879695
SMARCAD1	ENSP00000351947	ST	1	0	*no probe*	*no probe*	*no probe*	*no probe*	*no probe*	*no probe*	0.44053724	no	0.106229561
SOX10	ENSP00000354130	CON	4	0.00714344	0.105632401	no	0.746567393	0.004321	no	0.970404437	0.471768938	no	0.465272466

SOX2	ENSP00000323588	ST	11	0.00285805	-0.026059678	no	0.758192096	-0.13704	no	0.055856357	-1.424207065	no	0.205219819
SPHK1	ENSP00000313681	Ca	8	0.000928	0.332048987	**Up**	**0.022055475**	0.06938	no	0.192485996	-0.666475986	no	0.200180758
STAT3	ENSP00000264657	ST	28	0.0154567	-0.028899098	no	0.834520123	-0.09668	no	0.054324093	0.783935787	no	0.058644045
STIM1	ENSP00000300737	Ca	7	0.01353499	-0.244653986	**Down**	**0.000488812**	-0.03882	no	0.202125033	0.161127766	no	0.695734504
STIM2	ENSP00000417569	Ca	4	0.0000234	*no probe*	*no probe*	*no probe*	*no probe*	*no probe*	*no probe*	0.690876505	no	0.141103889
TACR1	ENSP00000303522	Ca	**42**	0.00000382	-0.126076241	no	0.124287544	-0.07838	no	0.140929089	-1.607489836	**Down**	**0.006947436**
TACR2	ENSP00000362403	Ca	**42**	0.00000382	-0.093732789	no	0.188585427	0.061091	no	0.208532278	0.25173582	no	0.46233114
TACR3	ENSP00000303325	Ca	**42**	0.00000382	0.094073756	no	0.196294259	0.037577	no	0.206309235	-0.01905164	no	0.902123229
TBX3	ENSP00000257566	ST	1	0	0.15667443	no	0.230868791	0.087755	no	0.46113262	1.014261146	no	0.09093181
TBXA2R	ENSP00000393333	Ca	**47**	0.00301697	-0.094549545	no	0.142307589	0.118784	**Up**	**0.003640564**	0.366669553	no	0.544911813
TCF3	ENSP00000262965	ST	18	**0.02573384**	0.030892143	no	0.803481248	0.118718	no	0.217139092	0.388196991	no	0.260191349
THY1	ENSP00000284240	ST	1	0	-0.473451414	**Down**	**0.000382645**	-0.25112	**Down**	**9.17744E-05**	-0.097689432	no	0.867512422
TLE4	ENSP00000365735	CON	5	0.00682854	0.130596856	no	0.449657637	0.017118	no	0.852363111	0.112191689	no	0.875586586
TRHR	ENSP00000309818	Ca	**42**	0.00000382	0.139025291	**Up**	**0.04514731**	0.110461	**Up**	**0.000240935**	-0.140199544	no	0.44240227
VDAC1	ENSP00000265333	Ca	7	0.01610604	0.314250026	**Up**	**0.018856803**	-0.00427	no	0.946198996	0.924120346	no	0.092489525
VDAC2	ENSP00000361635	Ca	4	0	0.100897002	no	0.478050986	-0.02276	no	0.620516463	-0.278891688	no	0.143231604
VDAC3	ENSP00000428845	Ca	5	0.00682594	0.122150714	no	0.313363466	0.040102	no	0.305878668	1.002343479	no	0.060285107
WNT1	ENSP00000293549	ST	33	0.01609896	-0.004445517	no	0.947027658	0.05291	no	0.138904226	-0.146432726	no	0.420289685
WNT10A	ENSP00000258411	ST	27	0.0000201	*no probe*	*no probe*	*no probe*	*no probe*	*no probe*	*no probe*	-1.051014506	**Down**	**0.006104193**
WNT10B	ENSP00000301061	ST	28	0.00423025	-0.065273366	no	0.549751248	-0.10201	no	0.172913743	-0.45959241	no	0.216414778
WNT11	ENSP00000325526	ST	27	0.0000201	-0.198779226	**Down**	**0.002237576**	-0.16037	**Down**	**0.02908149**	-0.60112241	no	0.126576731
WNT16	ENSP00000222462	ST	27	0.0000201	0.017443978	no	0.796308339	0.057444	no	0.237345562	-2.138890939	**Down**	**0.014976208**
WNT2	ENSP00000265441	ST	28	0.000774	0.149201682	no	0.077299817	0.103869	**Up**	**0.008500292**	-0.206964908	no	0.6597177
WNT2B	ENSP00000358698	ST	27	0.0000201	-0.05721198	no	0.393779725	0.012966	no	0.798007509	-0.290921771	no	0.404709278
WNT3	ENSP00000225512	ST	27	0.0000201	-0.345891879	**Down**	**9.14835E-07**	-0.08556	**Down**	**0.005379583**	-0.623606598	no	0.356300932
WNT3A	ENSP00000284523	ST	33	0.01580006	*no probe*	*no probe*	*no probe*	*no probe*	*no probe*	*no probe*	*no probe*	*no probe*	*no probe*
WNT4	ENSP00000290167	ST	27	0.0000201	-1.033784268	**Down**	**1.73126E-15**	-0.40809	**Down**	**7.82447E-15**	-1.477887308	**Down**	**0.017702159**
WNT5A	ENSP00000264634	ST	29	0.00866196	-0.625065857	**Down**	**2.03354E-06**	-0.22863	**Down**	**4.43848E-06**	-1.669255786	**Down**	**0.001785341**
WNT5B	ENSP00000308887	ST	27	0.0000201	-0.196621821	**Down**	**0.01713064**	-0.10938	no	0.058403641	-0.778666315	no	0.116573098
WNT6	ENSP00000233948	ST	27	0.0000201	0.089574217	no	0.323996843	-0.0946	**Down**	**0.030679811**	-0.595015336	no	0.128600012
WNT7A	ENSP00000285018	ST	28	0.000774	-0.079917014	no	0.116792267	-0.01928	no	0.689290889	-0.355790357	no	0.105068669
WNT7B	ENSP00000341032	ST	27	0.0000201	-0.312561559	**Down**	**4.87495E-06**	-0.19548	**Down**	**5.79645E-06**	-0.4638341	**Down**	**0.033164429**
WNT8A	ENSP00000381739	ST	30	0.00310753	*no probe*	*no probe*	*no probe*	*no probe*	*no probe*	*no probe*	-0.195474245	no	0.314072573
WNT8B	ENSP00000340677	ST	30	0.00310753	-0.11899919	no	0.086353151	-0.0183	no	0.778828694	-0.079045592	no	0.685834465
WNT9A	ENSP00000272164	ST	27	0.0000201	*no probe*	*no probe*	*no probe*	*no probe*	*no probe*	*no probe*	-0.796810057	**Down**	**0.017224254**

WNT9B	ENSP00000290015	ST	27	0.0000201	*no probe*	*no probe*	*no probe*	*no probe*	*no probe*	*no probe*	-0.066120029	no	0.696958394
ZIC3	ENSP00000287538	ST	5	0	0.120278857	no	0.065595744	0.151457	**Up**	**0.009490133**	-0.217487605	no	0.203226624
ZNF593	ENSP00000363384	ST	2	0.000164	-0.082583463	no	0.608328638	0.001154	no	0.984995034	0.422429503	no	0.441691105

Topology (Thresholds)

	Degree	*Betweenness*
**Mean**	20.37414966	6.56E-03
**SD**	16.8055374	0.012760705
**1 SD**	37.17968706	0.019322032
