# Supplementary material for: Network-Based Identification of Altered Stem Cell Pluripotency and Calcium Signaling Pathways in Metastatic Melanoma
Source: Med Sci (Basel). 2018 Mar 8;6(1):23. doi: 10.3390/medsci6010023 (PMC5872180; doi:10.3390/medsci6010023)
Supplement: Supplementary file 1 [file medsci-06-00023-s001.zip › Supplementary Table 1. Neves de Oliveira et al. 2018.pdf]

**Supplementary Table S1.** Genes/proteins belonging to the stem cell pluripotency (map04550) and calcium signaling (map04020) interaction network model ("STEMCa" network) and its gene subnetwork contributions (ST: stem cell pluripotency; $Ca^{2+}$: calcium signaling; CON: connector).

| Gene Symbol | Ensemble ID | Description | Subnetwork Contribution |
|---|---|---|---|
| *ACTB* | ENSP00000349960 | Actin, beta | CON |
| *ACVR1* | ENSP00000263640 | Activin A receptor, type I | ST |
| *ACVR2A* | ENSP00000241416 | Activin A receptor, type IIA | ST |
| *ACVR2B* | ENSP00000340361 | Activin A receptor, type IIB | ST |
| *ADCY1* | ENSP00000297323 | Adenylate cyclase 1 (brain) | Ca |
| *ADCY2* | ENSP00000342952 | Adenylate cyclase 2 (brain) | Ca |
| *ADCY3* | ENSP00000260600 | Adenylate cyclase 3 | Ca |
| *ADCY4* | ENSP00000312126 | Adenylate cyclase 4 | Ca |
| *ADCY7* | ENSP00000254235 | Adenylate cyclase 7 | Ca |
| *ADCY8* | ENSP00000286355 | Adenylate cyclase 8 (brain) | Ca |
| *ADCY9* | ENSP00000294016 | Adenylate cyclase 9 | Ca |
| *ADORA2A* | ENSP00000336630 | Adenosine A2a receptor | Ca |
| *ADORA2B* | ENSP00000304501 | Adenosine A2b receptor | Ca |
| *ADRA1A* | ENSP00000369960 | Adrenoceptor alpha 1A | Ca |
| *ADRA1B* | ENSP00000306662 | Adrenoceptor alpha 1B | Ca |
| *ADRA1D* | ENSP00000368766 | Adrenoceptor alpha 1D | Ca |
| *ADRB1* | ENSP00000358301 | Adrenoceptor beta 1 | Ca |
| *ADRB2* | ENSP00000305372 | Adrenoceptor beta 2 | Ca |
| *ADRB3* | ENSP00000343782 | Adrenoceptor beta 3 | Ca |
| *AGTR1* | ENSP00000273430 | Angiotensin II receptor, type 1 | Ca |
| *AKAP10* | ENSP00000225737 | A kinase (PRKA) anchor protein 10 | Ca |
| *AKT1* | ENSP00000270202 | v-akt murine thymoma viral oncogene homolog 1 | ST |
| *AKT2* | ENSP00000375892 | v-akt murine thymoma viral oncogene homolog 2 | ST |
| *AKT3* | ENSP00000263826 | v-akt murine thymoma viral oncogene homolog 3 (protein kinase B, gamma) | ST |
| *AKTIP* | ENSP00000378152 | AKT interacting protein | ST |
| *ALCAM* | ENSP00000305988 | Activated leukocyte cell adhesion molecule | ST |
| *ALDH1B1* | ENSP00000366927 | Aldehyde dehydrogenase 1 family, member B1 | ST |
| *ALDH3A1* | ENSP00000225740 | Aldehyde dehydrogenase 3 family, member A1 | ST |

| | | | |
|---|---|---|---|
| *APC* | ENSP00000257430 | Adenomatous polyposis coli | ST |
| *ARAF* | ENSP00000366244 | v-raf murine sarcoma 3611 viral oncogene homolog | ST |
| *ATP2A1* | ENSP00000349595 | ATPase, Ca++ transporting, cardiac muscle, fast twitch 1 | Ca |
| *ATP2A2* | ENSP00000440045 | ATPase, Ca++ transporting, cardiac muscle, slow twitch 2 | Ca |
| *AVP* | ENSP00000369647 | Arginine vasopressin | CON |
| *AVPR1A* | ENSP00000299178 | Arginine vasopressin receptor 1A | Ca |
| *AVPR1B* | ENSP00000356094 | Arginine vasopressin receptor 1B | Ca |
| *AXIN1* | ENSP00000262320 | Axin 1 | ST |
| *AXIN2* | ENSP00000302625 | Axin 2 | ST |
| *BDKRB1* | ENSP00000216629 | Bradykinin receptor B1 | Ca |
| *BDKRB2* | ENSP00000307713 | Bradykinin receptor B2 | Ca |
| *BMI1* | ENSP00000365851 | BMI1 polycomb ring finger oncogene | ST |
| *BMP2* | ENSP00000368104 | Bone morphogenetic protein 2 | ST |
| *BMP4* | ENSP00000245451 | Bone morphogenetic protein 4 | ST |
| *BMP6* | ENSP00000283147 | Bone morphogenetic protein 6 | ST |
| *BMP7* | ENSP00000379204 | Bone morphogenetic protein 7 | ST |
| *BMPR1A* | ENSP00000224764 | Bone morphogenetic protein receptor, type IA | ST |
| *BMPR1B* | ENSP00000264568 | Bone morphogenetic protein receptor, type IB | ST |
| *BMPR2* | ENSP00000363708 | Bone morphogenetic protein receptor, type II (serine/threonine kinase) | ST |
| *BRAF* | ENSP00000288602 | v-raf murine sarcoma viral oncogene homolog B1 | ST |
| *CACNA1A* | ENSP00000353362 | Calcium channel, voltage-dependent, P/Q type, alpha 1A subunit | Ca |
| *CACNA1B* | ENSP00000360406 | Calcium channel, voltage-dependent, N type, alpha 1B subunit | Ca |
| *CACNA1C* | ENSP00000266376 | Calcium channel, voltage-dependent, L type, alpha 1C subunit | Ca |
| *CACNA1D* | ENSP00000288139 | Calcium channel, voltage-dependent, L type, alpha 1D subunit | Ca |
| *CACNA1E* | ENSP00000356545 | Calcium channel, voltage-dependent, R type, alpha 1E subunit | Ca |
| *CACNA1F* | ENSP00000365441 | Calcium channel, voltage-dependent, L type, alpha 1F subunit | Ca |
| *CACNA1G* | ENSP00000352011 | Calcium channel, voltage-dependent, T type, alpha 1G subunit | Ca |
| *CACNA1H* | ENSP00000334198 | Calcium channel, voltage-dependent, T type, alpha 1H subunit | Ca |
| *CACNA1I* | ENSP00000385019 | Calcium channel, voltage-dependent, T type, alpha 1I subunit | Ca |
| *CACNA1S* | ENSP00000355192 | Calcium channel, voltage-dependent, L type, alpha 1S subunit | Ca |
| *CALM1* | ENSP00000349467 | Calmodulin 1 (phosphorylase kinase, delta) | CON |
| *CALM2* | ENSP00000272298 | Calmodulin 2 (phosphorylase kinase, delta) | Ca |
| *CAMK2B* | ENSP00000379098 | Calcium/calmodulin-dependent protein kinase II beta | Ca |

| | | | |
|---|---|---|---|
| *CAMK4* | ENSP00000282356 | Calcium/calmodulin-dependent protein kinase IV | Ca |
| *CCKAR* | ENSP00000295589 | Cholecystokinin A receptor | Ca |
| *CCKBR* | ENSP00000335544 | Cholecystokinin B receptor | Ca |
| *CCND1* | ENSP00000227507 | Cyclin D1 | CON |
| *CD38* | ENSP00000226279 | CD38 molecule | Ca |
| *CD44* | ENSP00000398632 | CD44 molecule (Indian blood group) | ST |
| *CD6* | ENSP00000323280 | CD6 molecule | ST |
| *CDH5* | ENSP00000344115 | Cadherin 5, type 2 (vascular endothelium) | CON |
| *CHRM1* | ENSP00000306490 | Cholinergic receptor, muscarinic 1 | Ca |
| *CHRM2* | ENSP00000319984 | Cholinergic receptor, muscarinic 2 | Ca |
| *CHRM3* | ENSP00000255380 | Cholinergic receptor, muscarinic 3 | Ca |
| *CHRM5* | ENSP00000372750 | Cholinergic receptor, muscarinic 5 | Ca |
| *CHRNA7* | ENSP00000407546 | Cholinergic receptor, nicotinic, alpha 7 (neuronal) | Ca |
| *CTNNA1* | ENSP00000304669 | Catenin (cadherin-associated protein), alpha 1, 102kDa | ST |
| *CTNNA2* | ENSP00000418191 | Catenin (cadherin-associated protein), alpha 2 | ST |
| *CTNNA3* | ENSP00000362849 | Catenin (cadherin-associated protein), alpha 3 | ST |
| *CTNNB1* | ENSP00000344456 | Catenin (cadherin-associated protein), beta 1, 88kDa | ST |
| *CTNNBIP1* | ENSP00000366466 | Catenin, beta interacting protein 1 | ST |
| *CTNND2* | ENSP00000307134 | Catenin (cadherin-associated protein), delta 2 | ST |
| *CYSLTR1* | ENSP00000362401 | Cysteinyl leukotriene receptor 1 | Ca |
| *CYSLTR2* | ENSP00000282018 | Cysteinyl leukotriene receptor 2 | Ca |
| *DLX5* | ENSP00000222598 | Distal-less homeobox 5 | ST |
| *DRD1* | ENSP00000327652 | Dopamine receptor D1 | Ca |
| *DRD5* | ENSP00000306129 | Dopamine receptor D5 | Ca |
| *DVL1* | ENSP00000368169 | Dishevelled, dsh homolog 1 (Drosophila) | ST |
| *EDNRA* | ENSP00000315011 | Endothelin receptor type A | Ca |
| *EDNRB* | ENSP00000366416 | Endothelin receptor type B | Ca |
| *EGFR* | ENSP00000275493 | Epidermal growth factor receptor | Ca |
| *ERBB2* | ENSP00000269571 | v-erb-b2 erythroblastic leukemia viral oncogene homolog 2, neuro/glioblastoma derived oncogene homolog (avian) | Ca |
| *ERBB3* | ENSP00000267101 | v-erb-b2 erythroblastic leukemia viral oncogene homolog 3 (avian) | Ca |
| *ERBB4* | ENSP00000342235 | v-erb-a erythroblastic leukemia viral oncogene homolog 4 (avian) | Ca |
| *ESR1* | ENSP00000206249 | Estrogen receptor 1 | CON |

| | | | |
|---|---|---|---|
| *ETV4* | ENSP00000321835 | Ets variant 4 | ST |
| *ETV5* | ENSP00000306894 | Ets variant 5 | ST |
| *F2R* | ENSP00000321326 | Coagulation factor II (thrombin) receptor | Ca |
| *FGF2* | ENSP00000264498 | Fibroblast growth factor 2 (basic) | ST |
| *FGFR1* | ENSP00000393312 | Fibroblast growth factor receptor 1 | ST |
| *FGFR2* | ENSP00000410294 | Fibroblast growth factor receptor 2 | ST |
| *FGFR3* | ENSP00000339824 | Fibroblast growth factor receptor 3 | ST |
| *FGFR4* | ENSP00000292408 | Fibroblast growth factor receptor 4 | ST |
| *FZD1* | ENSP00000287934 | Frizzled family receptor 1 | ST |
| *FZD10* | ENSP00000229030 | Frizzled family receptor 10 | ST |
| *FZD2* | ENSP00000323901 | Frizzled family receptor 2 | ST |
| *FZD3* | ENSP00000240093 | Frizzled family receptor 3 | ST |
| *FZD4* | ENSP00000434034 | Frizzled family receptor 4 | ST |
| *FZD5* | ENSP00000354607 | Frizzled family receptor 5 | ST |
| *FZD6* | ENSP00000351605 | Frizzled family receptor 6 | ST |
| *FZD7* | ENSP00000286201 | Frizzled family receptor 7 | ST |
| *FZD8* | ENSP00000363826 | Frizzled family receptor 8 | ST |
| *FZD9* | ENSP00000345785 | Frizzled family receptor 9 | ST |
| *GABRR1* | ENSP00000412673 | Gamma-aminobutyric acid (GABA) A receptor, rho 1 | CON |
| *GDF5* | ENSP00000363489 | Growth differentiation factor 5 | ST |
| *GNA11* | ENSP00000078429 | Guanine nucleotide binding protein (G protein), alpha 11 (Gq class) | Ca |
| *GNA14* | ENSP00000365807 | Guanine nucleotide binding protein (G protein), alpha 14 | Ca |
| *GNA15* | ENSP00000262958 | Guanine nucleotide binding protein (G protein), alpha 15 (Gq class) | Ca |
| *GNAL* | ENSP00000334051 | Guanine nucleotide binding protein (G protein), alpha activating activity polypeptide, olfactory type | Ca |
| *GNAQ* | ENSP00000286548 | Guanine nucleotide binding protein (G protein), q polypeptide | Ca |
| *GNAS* | ENSP00000360141 | GNAS complex locus | Ca |
| *GRB2* | ENSP00000339007 | Growth factor receptor-bound protein 2 | ST |
| *GRIN1* | ENSP00000360608 | Glutamate receptor, ionotropic, N-methyl D-aspartate 1 | Ca |
| *GRIN2A* | ENSP00000332549 | Glutamate receptor, ionotropic, N-methyl D-aspartate 2A | Ca |
| *GRIN2C* | ENSP00000293190 | Glutamate receptor, ionotropic, N-methyl D-aspartate 2C | Ca |
| *GRIN2D* | ENSP00000263269 | Glutamate receptor, ionotropic, N-methyl D-aspartate 2D | Ca |
| *GRM1* | ENSP00000282753 | Glutamate receptor, metabotropic 1 | Ca |
| *GRM5* | ENSP00000306138 | Glutamate receptor, metabotropic 5 | Ca |

| | | | |
|---|---|---|---|
| *GRPR* | ENSP00000369643 | Gastrin-releasing peptide receptor | Ca |
| *GSK3B* | ENSP00000324806 | Glycogen synthase kinase 3 beta | ST |
| *GSTP1* | ENSP00000381607 | Glutathione S-transferase pi 1 | CON |
| *HAND1* | ENSP00000231121 | Heart and neural crest derivatives expressed 1 | ST |
| *HDAC1* | ENSP00000362649 | Histone deacetylase 1 | CON |
| *HESX1* | ENSP00000295934 | HESX homeobox 1 | ST |
| *HOXB1* | ENSP00000355140 | Homeobox B1 | ST |
| *HRAS* | ENSP00000309845 | v-Ha-ras Harvey rat sarcoma viral oncogene homolog | ST |
| *HRH1* | ENSP00000380247 | Histamine receptor H1 | Ca |
| *HRH2* | ENSP00000366506 | Histamine receptor H2 | Ca |
| *HTR2A* | ENSP00000367959 | 5-hydroxytryptamine (serotonin) receptor 2A, G protein-coupled | Ca |
| *HTR4* | ENSP00000353915 | 5-hydroxytryptamine (serotonin) receptor 4, G protein-coupled | Ca |
| *HTR6* | ENSP00000289753 | 5-hydroxytryptamine (serotonin) receptor 6, G protein-coupled | Ca |
| *HTR7* | ENSP00000337949 | 5-hydroxytryptamine (serotonin) receptor 7, adenylate cyclase-coupled | Ca |
| *ID1* | ENSP00000365280 | Inhibitor of DNA binding 1, dominant negative helix-loop-helix protein | ST |
| *ID2* | ENSP00000234091 | Inhibitor of DNA binding 2, dominant negative helix-loop-helix protein | ST |
| *ID3* | ENSP00000363689 | Inhibitor of DNA binding 3, dominant negative helix-loop-helix protein | ST |
| *ID4* | ENSP00000367972 | Inhibitor of DNA binding 4, dominant negative helix-loop-helix protein | ST |
| *IGF1* | ENSP00000302665 | Insulin-like growth factor 1 (somatomedin C) | CON |
| *IGFBP1* | ENSP00000275525 | Insulin-like growth factor binding protein 1 | ST |
| *IGFBP2* | ENSP00000233809 | Insulin-like growth factor binding protein 2, 36kDa | ST |
| *IGFBP3* | ENSP00000370473 | Insulin-like growth factor binding protein 3 | ST |
| *IGFBP4* | ENSP00000269593 | Insulin-like growth factor binding protein 4 | ST |
| *IGFBP5* | ENSP00000233813 | Insulin-like growth factor binding protein 5 | ST |
| *IGFBP6* | ENSP00000301464 | Insulin-like growth factor binding protein 6 | ST |
| *IGFBP7* | ENSP00000295666 | Insulin-like growth factor binding protein 7 | ST |
| *ISL1* | ENSP00000230658 | ISL LIM homeobox 1 | ST |
| *ITPR1* | ENSP00000306253 | Inositol 1,4,5-trisphosphate receptor, type 1 | Ca |
| *ITPR2* | ENSP00000370744 | Inositol 1,4,5-trisphosphate receptor, type 2 | Ca |
| *ITPR3* | ENSP00000363435 | Inositol 1,4,5-trisphosphate receptor, type 3 | Ca |
| *JARID2* | ENSP00000341280 | Jumonji, AT rich interactive domain 2 | ST |
| *JUN* | ENSP00000360266 | Jun proto-oncogene | CON |
| *KAT2B* | ENSP00000263754 | K(lysine) acetyltransferase 2B | CON |

| | | | |
|---|---|---|---|
| *KAT6A* | ENSP00000265713 | K(lysine) acetyltransferase 6A | ST |
| *KIT* | ENSP00000288135 | v-kit Hardy-Zuckerman 4 feline sarcoma viral oncogene homolog | ST |
| *KLF4* | ENSP00000363804 | Kruppel-like factor 4 (gut) | ST |
| *LEF1* | ENSP00000265165 | Lymphoid enhancer-binding factor 1 | ST |
| *LEFTY2* | ENSP00000355785 | Left-right determination factor 2 | ST |
| *LHCGR* | ENSP00000294954 | Luteinizing hormone/choriogonadotropin receptor | Ca |
| *LIF* | ENSP00000249075 | Leukemia inhibitory factor | ST |
| *LIFR* | ENSP00000263409 | Leukemia inhibitory factor receptor alpha | ST |
| *LTB4R2* | ENSP00000433290 | Leukotriene B4 receptor 2 | Ca |
| *LYN* | ENSP00000428924 | v-yes-1 Yamaguchi sarcoma viral related oncogene homolog | ST |
| *MAOA* | ENSP00000340684 | Monoamine oxidase A | CON |
| *MAP3K1* | ENSP00000382423 | Mitogen-activated protein kinase kinase kinase 1, E3 ubiquitin protein ligase | ST |
| *MAPK1* | ENSP00000215832 | Mitogen-activated protein kinase 1 | ST |
| *MAPK11* | ENSP00000333685 | Mitogen-activated protein kinase 11 | ST |
| *MAPK12* | ENSP00000215659 | Mitogen-activated protein kinase 12 | ST |
| *MAPK13* | ENSP00000211287 | Mitogen-activated protein kinase 13 | ST |
| *MAPK14* | ENSP00000229794 | Mitogen-activated protein kinase 14 | ST |
| *MAPK3* | ENSP00000263025 | Mitogen-activated protein kinase 3 | ST |
| *MEIS1* | ENSP00000272369 | Meis homeobox 1 | ST |
| *MSC* | ENSP00000321445 | Musculin | ST |
| *MYC* | ENSP00000367207 | v-myc myelocytomatosis viral oncogene homolog (avian) | ST |
| *MYLK* | ENSP00000353452 | Myosin light chain kinase | Ca |
| *NANOG* | ENSP00000229307 | Nanog homeobox | ST |
| *NANOGP1* | ENSP00000432545 | Nanog homeobox pseudogene 1 | ST |
| *NCOR1* | ENSP00000268712 | Nuclear receptor corepressor 1 | CON |
| *NEUROG1* | ENSP00000317580 | Neurogenin 1 | ST |
| *NHP2* | ENSP00000274606 | NHP2 ribonucleoprotein homolog (yeast) | CON |
| *NODAL* | ENSP00000287139 | Nodal homolog (mouse) | ST |
| *NOS1* | ENSP00000337459 | Nitric oxide synthase 1 (neuronal) | Ca |
| *NOS2* | ENSP00000327251 | Nitric oxide synthase 2, inducible | Ca |
| *NOS3* | ENSP00000297494 | Nitric oxide synthase 3 (endothelial cell) | Ca |
| *NRAS* | ENSP00000358548 | Neuroblastoma RAS viral (v-ras) oncogene homolog | ST |
| *NTSR1* | ENSP00000359532 | Neurotensin receptor 1 (high affinity) | Ca |

| | | | |
|---|---|---|---|
| *ONECUT1* | ENSP00000302630 | One cut homeobox 1 | ST |
| *ORAI1* | ENSP00000328216 | ORAI calcium release-activated calcium modulator 1 | Ca |
| *ORAI2* | ENSP00000348752 | ORAI calcium release-activated calcium modulator 2 | Ca |
| *ORAI3* | ENSP00000322249 | ORAI calcium release-activated calcium modulator 3 | Ca |
| *OTX1* | ENSP00000282549 | Orthodenticle homeobox 1 | ST |
| *OXTR* | ENSP00000324270 | Oxytocin receptor | Ca |
| *P2RX1* | ENSP00000225538 | Purinergic receptor P2X, ligand-gated ion channel, 1 | Ca |
| *P2RX2* | ENSP00000343339 | Purinergic receptor P2X, ligand-gated ion channel, 2 | Ca |
| *P2RX3* | ENSP00000263314 | Purinergic receptor P2X, ligand-gated ion channel, 3 | Ca |
| *P2RX4* | ENSP00000336607 | Purinergic receptor P2X, ligand-gated ion channel, 4 | Ca |
| *P2RX6* | ENSP00000416193 | Purinergic receptor P2X, ligand-gated ion channel, 6 | Ca |
| *P2RX7* | ENSP00000442349 | Purinergic receptor P2X, ligand-gated ion channel, 7 | Ca |
| *PAX6* | ENSP00000368401 | Paired box 6 | ST |
| *PDGFRA* | ENSP00000257290 | Platelet-derived growth factor receptor, alpha polypeptide | Ca |
| *PDGFRB* | ENSP00000261799 | Platelet-derived growth factor receptor, beta polypeptide | Ca |
| *PHKG1* | ENSP00000297373 | Phosphorylase kinase, gamma 1 (muscle) | Ca |
| *PIK3CG* | ENSP00000352121 | Phosphatidylinositol-4,5-bisphosphate 3-kinase, catalytic subunit gamma | ST |
| *PIK3R1* | ENSP00000274335 | Phosphoinositide-3-kinase, regulatory subunit 1 (alpha) | ST |
| *PIK3R5* | ENSP00000392812 | Phosphoinositide-3-kinase, regulatory subunit 5 | ST |
| *PLCB1* | ENSP00000338185 | Phospholipase C, beta 1 (phosphoinositide-specific) | Ca |
| *PLCB2* | ENSP00000260402 | Phospholipase C, beta 2 | Ca |
| *PLCB3* | ENSP00000279230 | Phospholipase C, beta 3 (phosphatidylinositol-specific) | Ca |
| *PLCB4* | ENSP00000334105 | Phospholipase C, beta 4 | Ca |
| *PLCD1* | ENSP00000430344 | Phospholipase C, delta 1 | Ca |
| *PLCD3* | ENSP00000313731 | Phospholipase C, delta 3 | Ca |
| *PLCD4* | ENSP00000388631 | Phospholipase C, delta 4 | Ca |
| *PLCE1* | ENSP00000260766 | Phospholipase C, epsilon 1 | Ca |
| *PLCG1* | ENSP00000244007 | Phospholipase C, gamma 1 | Ca |
| *PLN* | ENSP00000350132 | Phospholamban | Ca |
| *POU5F1* | ENSP00000259915 | POU class 5 homeobox 1 | ST |
| *PPIF* | ENSP00000225174 | Peptidylprolyl isomerase F | Ca |
| *PPP1CC* | ENSP00000335084 | Protein phosphatase 1, catalytic subunit, gamma isozyme | CON |
| *PRC1* | ENSP00000377793 | Protein regulator of cytokinesis 1 | ST |

| | | | |
|---|---|---|---|
| *PRKACA* | ENSP00000309591 | Protein kinase, cAMP-dependent, catalytic, alpha | CON |
| *PRKCA* | ENSP00000408695 | Protein kinase C, alpha | Ca |
| *PRKCB* | ENSP00000305355 | Protein kinase C, beta | Ca |
| *PRKCG* | ENSP00000263431 | Protein kinase C, gamma | Ca |
| *PROM1* | ENSP00000415481 | Prominin 1 | ST |
| *PTAFR* | ENSP00000301974 | Platelet-activating factor receptor | Ca |
| *PTEN* | ENSP00000361021 | Phosphatase and tensin homolog | ST |
| *PTGER1* | ENSP00000292513 | Prostaglandin E receptor 1 (subtype EP1), 42kDa | Ca |
| *PTGER3* | ENSP00000349003 | Prostaglandin E receptor 3 (subtype EP3) | Ca |
| *PTGFR* | ENSP00000359793 | Prostaglandin F receptor (FP) | Ca |
| *PTK2B* | ENSP00000332816 | PTK2B protein tyrosine kinase 2 beta | Ca |
| *PTPN11* | ENSP00000340944 | Protein tyrosine phosphatase, non-receptor type 11 | ST |
| *RAF1* | ENSP00000251849 | v-raf-1 murine leukemia viral oncogene homolog 1 | ST |
| *REST* | ENSP00000311816 | RE1-silencing transcription factor | ST |
| *RIF1* | ENSP00000243326 | RAP1 interacting factor homolog (yeast) | ST |
| *RRAS* | ENSP00000246792 | Related RAS viral (r-ras) oncogene homolog | ST |
| *RYR1* | ENSP00000352608 | Ryanodine receptor 1 (skeletal) | Ca |
| *RYR2* | ENSP00000355533 | Ryanodine receptor 2 (cardiac) | Ca |
| *RYR3* | ENSP00000373884 | Ryanodine receptor 3 | Ca |
| *SALL4* | ENSP00000217086 | Sal-like 4 (Drosophila) | CON |
| *SDC1* | ENSP00000254351 | Syndecan 1 | CON |
| *SETDB1* | ENSP00000271640 | SET domain, bifurcated 1 | ST |
| *SKIL* | ENSP00000259119 | SKI-like oncogene | ST |
| *SLC25A4* | ENSP00000281456 | Solute carrier family 25 (mitochondrial carrier; adenine nucleotide translocator), member 4 | Ca |
| *SMAD2* | ENSP00000262160 | SMAD family member 2 | ST |
| *SMAD3* | ENSP00000332973 | SMAD family member 3 | ST |
| *SMAD4* | ENSP00000341551 | SMAD family member 4 | ST |
| *SMAD6* | ENSP00000288840 | SMAD family member 6 | ST |
| *SMAD7* | ENSP00000262158 | SMAD family member 7 | ST |
| *SMARCAD1* | ENSP00000351947 | SWI/SNF-related, matrix-associated actin-dependent regulator of chromatin, subfamily a, containing DEAD/H box 1 | ST |
| *SOX10* | ENSP00000354130 | SRY (sex determining region Y)-box 10 | CON |
| *SOX2* | ENSP00000323588 | SRY (sex determining region Y)-box 2 | ST |

| | | | |
|---|---|---|---|
| *SPHK1* | ENSP00000313681 | Sphingosine kinase 1 | Ca |
| *STAT3* | ENSP00000264657 | Signal transducer and activator of transcription 3 (acute-phase response factor) | ST |
| *STIM1* | ENSP00000300737 | Stromal interaction molecule 1 | Ca |
| *STIM2* | ENSP00000417569 | Stromal interaction molecule 2 | Ca |
| *TACR1* | ENSP00000303522 | Tachykinin receptor 1 | Ca |
| *TACR2* | ENSP00000362403 | Tachykinin receptor 2 | Ca |
| *TACR3* | ENSP00000303325 | Tachykinin receptor 3 | Ca |
| *TBX3* | ENSP00000257566 | T-box 3 | ST |
| *TBXA2R* | ENSP00000393333 | Thromboxane A2 receptor | Ca |
| *TCF3* | ENSP00000262965 | Transcription factor 3 (E2A immunoglobulin enhancer binding factors E12/E47) | ST |
| *THY1* | ENSP00000284240 | Thy-1 cell surface antigen | ST |
| *TLE4* | ENSP00000365735 | Transducin-like enhancer of split 4 (E(sp1) homolog, Drosophila) | CON |
| *TRHR* | ENSP00000309818 | Thyrotropin-releasing hormone receptor | Ca |
| *VDAC1* | ENSP00000265333 | Voltage-dependent anion channel 1 | Ca |
| *VDAC2* | ENSP00000361635 | Voltage-dependent anion channel 2 | Ca |
| *VDAC3* | ENSP00000428845 | Voltage-dependent anion channel 3 | Ca |
| *WNT1* | ENSP00000293549 | Wingless-type MMTV integration site family, member 1 | ST |
| *WNT10A* | ENSP00000258411 | Wingless-type MMTV integration site family, member 10A | ST |
| *WNT10B* | ENSP00000301061 | Wingless-type MMTV integration site family, member 10B | ST |
| *WNT11* | ENSP00000325526 | Wingless-type MMTV integration site family, member 11 | ST |
| *WNT16* | ENSP00000222462 | Wingless-type MMTV integration site family, member 16 | ST |
| *WNT2* | ENSP00000265441 | Wingless-type MMTV integration site family member 2 | ST |
| *WNT2B* | ENSP00000358698 | Wingless-type MMTV integration site family, member 2B | ST |
| *WNT3* | ENSP00000225512 | Wingless-type MMTV integration site family, member 3 | ST |
| *WNT3A* | ENSP00000284523 | Wingless-type MMTV integration site family, member 3A | ST |
| *WNT4* | ENSP00000290167 | Wingless-type MMTV integration site family, member 4 | ST |
| *WNT5A* | ENSP00000264634 | Wingless-type MMTV integration site family, member 5A | ST |
| *WNT5B* | ENSP00000308887 | Wingless-type MMTV integration site family, member 5B | ST |
| *WNT6* | ENSP00000233948 | Wingless-type MMTV integration site family, member 6 | ST |
| *WNT7A* | ENSP00000285018 | Wingless-type MMTV integration site family, member 7A | ST |
| *WNT7B* | ENSP00000341032 | Wingless-type MMTV integration site family, member 7B | ST |
| *WNT8A* | ENSP00000381739 | Wingless-type MMTV integration site family, member 8A | ST |
| *WNT8B* | ENSP00000340677 | Wingless-type MMTV integration site family, member 8B | ST |

| | | | |
|---|---|---|---|
| *WNT9A* | ENSP00000272164 | Wingless-type MMTV integration site family, member 9A | ST |
| *WNT9B* | ENSP00000290015 | Wingless-type MMTV integration site family, member 9B | ST |
| *ZIC3* | ENSP00000287538 | Zic family member 3 | ST |
| *ZNF593* | ENSP00000363384 | Zinc finger protein 593 | ST |
